# Delivery of crop pollination services is an insufficient argument for wild pollinator conservation

**DOI:** 10.1038/ncomms8414

**Published:** 2015-06-16

**Authors:** David Kleijn, Rachael Winfree, Ignasi Bartomeus, Luísa G Carvalheiro, Mickaël Henry, Rufus Isaacs, Alexandra-Maria Klein, Claire Kremen, Leithen K M'Gonigle, Romina Rader, Taylor H Ricketts, Neal M Williams, Nancy Lee Adamson, John S Ascher, András Báldi, Péter Batáry, Faye Benjamin, Jacobus C Biesmeijer, Eleanor J Blitzer, Riccardo Bommarco, Mariëtte R Brand, Vincent Bretagnolle, Lindsey Button, Daniel P Cariveau, Rémy Chifflet, Jonathan F Colville, Bryan N Danforth, Elizabeth Elle, Michael P.D. Garratt, Felix Herzog, Andrea Holzschuh, Brad G Howlett, Frank Jauker, Shalene Jha, Eva Knop, Kristin M Krewenka, Violette Le Féon, Yael Mandelik, Emily A May, Mia G Park, Gideon Pisanty, Menno Reemer, Verena Riedinger, Orianne Rollin, Maj Rundlöf, Hillary S Sardiñas, Jeroen Scheper, Amber R Sciligo, Henrik G Smith, Ingolf Steffan-Dewenter, Robbin Thorp, Teja Tscharntke, Jort Verhulst, Blandina F Viana, Bernard E Vaissière, Ruan Veldtman, Catrin Westphal, Simon G Potts

**Affiliations:** 1 Animal Ecology Team, Center for Ecosystem Studies, Alterra, Wageningen UR, PO Box 47, 6700AA Wageningen, The Netherlands; 2 Resource Ecology Group, Wageningen University, Droevendaalsesteeg 3a, 6708 PB Wageningen, The Netherlands; 3 Department of Ecology, Evolution and Natural Resources, Rutgers University, 14 College Farm Road, New Brunswick, New Jersey 08901, USA; 4 Departmento Ecología Integrativa, Estación Biológica de Doñana (EDB-CSIC), Avenida Américo Vespucio s/n, 41092 Sevilla, Spain; 5 School of Biology, University of Leeds, Miall Building, Clarendon Road, Leeds LS2 9JT, UK; 6 Department of Terrestrial Zoology, Naturalis Biodiversity Center, PO Box 9517, 2300 RA Leiden, The Netherlands; 7 UR 406 Abeilles et Environnement, INRA, CS 40509, F-84914 Avignon, France; 8 UMT Protection des Abeilles dans l'Environnement, INRA, CS 40509, F-84914 Avignon, France; 9 Department of Entomology, Michigan State University, 578 Wilson Road, East Lansing, Michigan 48824, USA; 10 Nature Conservation and Landscape Ecology Group, Earth and Environmental Sciences, University of Freiburg, Freiburg D-79106, Germany; 11 Department of Environmental Science, Policy and Management, University of California, 130 Mulford Hall, Berkeley, California 94720-3114, USA; 12 School of Environmental and Rural Science, University of New England, Armidale, New South Wales 2350, Australia; 13 Gund Institute for Ecological Economics, University of Vermont, 617 Main Street, Burlington, Vermont 05405, USA; 14 Department of Entomology and Nematology, University of California, Davis, 1 Shields Avenue, Davis, California 95616, USA; 15 PO Box 20653, Greensboro, North Carolina 27420, USA; 16 Department of Biological Sciences, National University of Singapore, 14 Science Drive 4, Singapore 117543, Singapore; 17 Institute of Ecology and Botany, MTA Centre for Ecological Research, Alkotmány u. 2-4, Vácrátót 2163, Hungary; 18 Agroecology Group, Department of Crop Sciences, Georg-August-University, Grisebachstr. 6, 37077 Göttingen, Germany; 19 Department of Entomology, Cornell University, Ithaca, New York 14853, USA; 20 Department of Ecology, Swedish University of Agricultural Sciences, Uppsala 75007, Sweden; 21 South African National Biodiversity Institute, Kirstenbosch Research Centre, Private Bag X7, Claremont 7735, South Africa; 22 Conservation Ecology and Entomology, Stellenbosch University, Private Bag X1, Matieland 7602, South Africa; 23 Iziko South African Museum, 25 Queen Victoria Street, Cape Town 8000, South Africa; 24 Centre d'Etudes Biologiques de Chizé, UMR 7372, CNRS and Université La Rochelle, F-79360 Beauvoir-sur-Niort, France; 25 Department of Biological Sciences, Simon Fraser University,8888 University Drive, Burnaby, British Columbia, Canada V5A 1S6; 26 Plateforme Régionale d'Innovation "Agriculture Biologique et Périurbaine Durable", EPLEFPA du Lycée Nature, Allée des Druides, 85000 La Roche-sur-Yon, France; 27 Centre for Agri-Environmental Research, School of Agriculture, Policy and Development, University of Reading, Reading RG6 6AR, UK; 28 Agricultural Landscapes and Biodiversity, Agroscope, Reckenholzstr. 191, CH-8046 Zurich, Switzerland; 29 Department of Animal Ecology and Tropical Biology, Biocenter, University of Würzburg, Am Hubland, 97074 Würzburg, Germany; 30 Sustainable Production, The New Zealand Institute for Plant and Food Research Limited, Private Bag 4704, Christchurch 8140, New Zealand; 31 Department of Animal Ecology, Justus Liebig University Giessen, Heinrich-Buff-Ring 26-32, D-35392 Giessen, Germany; 32 Department of Integrative Biology, University of Texas at Austin, 401 Biological Laboratories, Austin, Texas 78712, USA; 33 Community Ecology Group, University of Bern, Baltzerstr. 6, 3012 Bern, Switzerland; 34 Department of Entomology, The Hebrew University of Jerusalem, PO Box 12, Rehovot 76100, Israel; 35 EIS Kenniscentrum Insecten, Naturalis Biodiversity Center, PO Box 9517, 2300 RA Leiden, The Netherlands; 36 ITSAP – Institut de l'abeille, 149 rue de Bercy, F-75012 Paris, France; 37 Department of Biology, Lund University, S-223 62 Lund, Sweden; 38 Centre of Environmental and Climate Research, Lund University, S-223 62 Lund, Sweden; 39 Spotvogellaan 68, 2566 PN Den Haag, The Netherlands; 40 Biology Institute, Federal University of Bahia, Rua Barão de Jeremoabo, s/n, Campus Universitário de Ondina, Salvador, Bahia 40170-290, Brazil

## Abstract

There is compelling evidence that more diverse ecosystems deliver greater benefits to people, and these ecosystem services have become a key argument for biodiversity conservation. However, it is unclear how much biodiversity is needed to deliver ecosystem services in a cost-effective way. Here we show that, while the contribution of wild bees to crop production is significant, service delivery is restricted to a limited subset of all known bee species. Across crops, years and biogeographical regions, crop-visiting wild bee communities are dominated by a small number of common species, and threatened species are rarely observed on crops. Dominant crop pollinators persist under agricultural expansion and many are easily enhanced by simple conservation measures, suggesting that cost-effective management strategies to promote crop pollination should target a different set of species than management strategies to promote threatened bees. Conserving the biological diversity of bees therefore requires more than just ecosystem-service-based arguments.

## Abstract (web summary)

One argument for conserving biological diversity is that it delivers beneficial ecosystem services. However, Kleijn *et al.* show that the economic benefits of crop pollination are delivered by only a small subset of relatively common species, arguing that threatened species should be considered separately.

## Graphical abstract

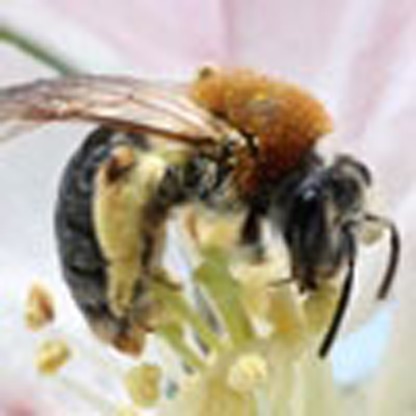

Worldwide, biodiversity is declining at unprecedented rates, threatening species persistence as well as the benefits humans gain from ecosystems^1^,^2^,^3^. These benefits, known as ecosystem services, have become an increasingly important argument for biodiversity conservation^4^,^5^,^6^,^7^,^8^. The economic and other benefits from ecosystems can motivate conservation action, and are more and more being used in payment for ecosystem service schemes. Once an economic value of the service has been determined, it can be captured in commercial markets or quantified in terms comparable with economic services and manufactured capital^9^. These economic values can then potentially be used to support biodiversity conservation within policies.

The use of ecosystem services arguments for justifying biodiversity conservation is, however, not without risk or controversy. Many experimental studies show that biodiversity increases the magnitude and/or stability of ecosystem functioning (of which ecosystem services are the subset that benefit people), and that most species contribute to ecosystem functioning in some way^10^,^11^,^12^,^13^. However, such studies do not consider the costs of maintaining or promoting biodiversity, even though costs are generally a limiting factor for implementing real-world conservation policies^14^. When the economic pay-off from ecosystem services is the main factor motivating conservation, the cost-effective action is to conserve the subset of species that provide the greatest return at relatively short timescales. Because real-world communities are almost invariably dominated by a small number of species^15^,^16^ that often respond readily to conservation management^17^, we hypothesize that in real-world landscapes (1) the majority of the services is provided by a relatively small number of species; (2) that these species are generally common, and that threatened species rarely contribute to present ecosystem service delivery; and (3) that the most important ecosystem-service-providing species can be easily enhanced by simple management actions that are insufficient to support threatened species. Support for these hypotheses would suggest that delivery of ecosystem services is insufficient as a general argument for biodiversity conservation^18^,^19^,^20^,^21^.

Here we test these hypotheses using data from 90 studies and 1,394 crop fields on crop-visiting bee communities from five continents. Pollination is an important ecosystem service. The economic contribution of pollinators to crop production is significant^22^, and bees are generally considered the most important pollinators of crops^23^. We find that wild bee communities contribute on average over $3,000 ha^−1^ to the production of insect-pollinated crops. However, a limited subset of all known bee species provides the majority of pollination services because, across different crops, years and large biogeographical regions, crop-visiting bee communities are dominated by a small number of common species and rarely contain regionally threatened species. Dominant crop pollinators are furthermore able to persist under agricultural expansion and many are relatively easily enhanced by simple conservation measures. Focusing conservation on the services delivered by pollinators may therefore lead to management strategies that predominantly benefit the limited set of species currently providing the majority of crop pollination. Consequently, conservation of the biological diversity of bees should be motivated not only by immediate benefits from ecosystem services but also by the full richness of arguments for conservation.

## Results

### The crop production value of wild bees

**Figure 1:**
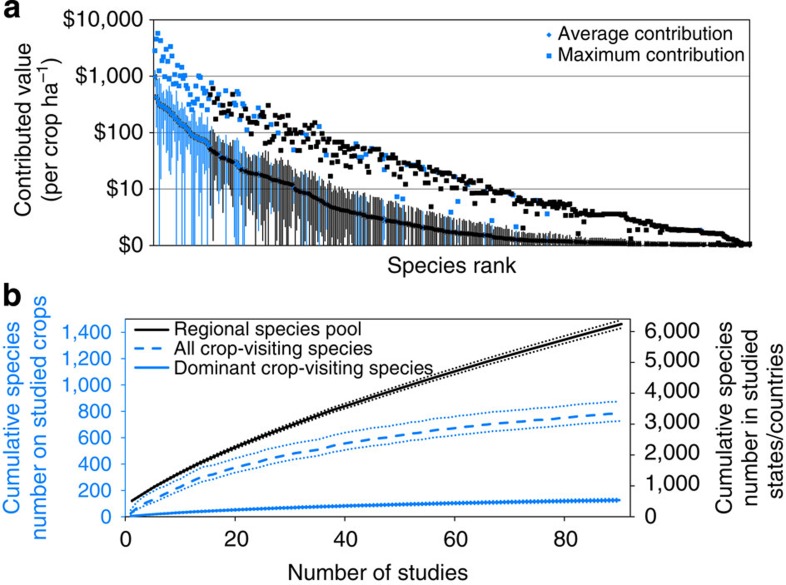
The relative contribution of individual species in wild bee communities to crop pollination. (**a**) The rank distribution of the contribution of wild bee species to crop production value in their biogeographical area. Dominant species, contributing at least 5% of all visits within a given study, are indicated in blue. Bars indicate 95% confidence intervals. (**b**) The cumulative number of bee species known to exist in the countries in which the studies were done, compared with an asymptotic estimate of the number of species that visit the flowers of the studied crops (Chao1 estimator), and the number of dominant crop-visiting wild bee species. Lightly dashed lines indicate estimates±s.e.

On average, wild bee communities contributed $3,251 ha^−1^ to production of the examined crops (s.e.=$547, range $7–14,252), about the same as the contribution of managed honey bees (mean±s.e.=$2,913±574, range $0–18,679). Individual wild bee species contribute substantially to crop production value with contributions up to $963 per crop ha^−1^ per species (mean across studies; Fig. 1a). Twenty-five species have a mean contribution higher than $100 ha^−1^ and 93 species have a maximum contribution higher than $100 ha^-1^ (Supplementary Table 2). The maximum contributions were 16.0 (±0.34) times higher than the mean contributions, suggesting that for most species large contributions to pollination are limited to specific years, crops and/or sites.

### The proportion of bee species contributing to pollination

Figure 1a also suggests that a small number of species dominate the contribution of wild bees to crop production value. Across the 90 studies, we collected a total of 73,649 individual bees of 785 species visiting crop flowers. Although this is an impressive number, it represents only 12.6% of the currently known number of species occurring in the states or countries where our studies took place (Fig. 1b). When we consider only bee species that contribute 5% or more to the relative visitation rate of any single study (hereafter, dominant crop-visiting species), the percentage drops to 2.0% of the species in the regional species pool (Fig. 1b). Yet these 2% of species account for almost 80% of all crop visits (Supplementary Fig. 2). The gentle slope of the species accumulation curve in Fig. 1b suggests that there is little turnover in dominant crop-visiting species between years, crops and locations, mainly because within biogeographical regions, a small number of species tend to dominate the crop-visiting bee communities everywhere (Supplementary Table 2).

### The commonness of crop-visiting bee species

To test the hypothesis that the species providing the majority of the pollination services are generally regionally common species, we use two lines of enquiry. First, we examined the contribution of threatened bee species to the set of bee species found on crops. Four of the countries we studied have compiled Red Data books for bees, which we used to objectively identify threatened species. In these countries, on average 44% of the bee species are threatened, but in the 19 studies carried out in these countries only 12 threatened species were found accounting for 0.3% (s.e. 0.1%) of the individual bees observed on crops. Second, we determined whether the dominant crop-visiting bee species are common in agricultural landscapes generally, using an independent data set of bee communities in 264 sites in agricultural landscapes in Europe and North America (see Methods section). These studies compared bee communities in agricultural habitats such as arable fields (but not flowering, bee-pollinated crops), grasslands, old fields and hedgerows with bee communities in nearby sites that are actively managed for biodiversity enhancement (for example, agri-environment schemes and wildflower plantings) (Supplementary Fig. 1; refs 17, 24). We used only the agricultural habitat controls to evaluate the frequency of dominant crop-visiting bee species (listed in Supplementary Table 3) in these ‘background' agricultural habitats.

**Figure 2:**
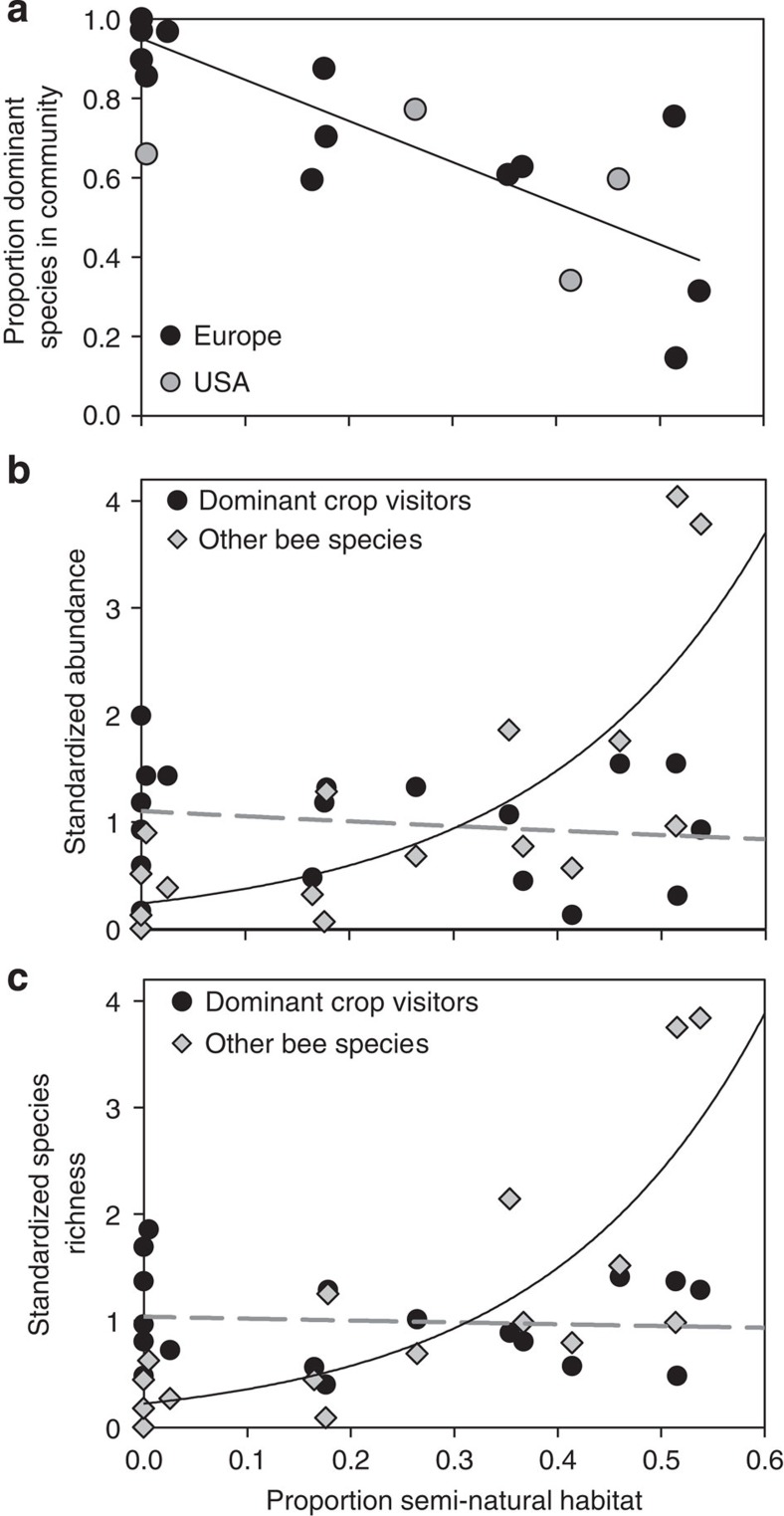
The relation between dominant crop-visiting bee species and cover of semi-natural habitats in agricultural landscapes. (**a**) The proportion of dominant crop-visiting bee species in bee communities in habitats other than flowering crops is negatively related to the proportion of semi-natural habitat within a 1,000-m radius (*F*_1,14_=14.47, *P*=0.002). (**b**) The relation between the proportion of semi-natural habitat in agricultural landscapes and bee abundance differs between dominant crop-visiting species and all other bee species (interaction type of bee and cover semi-natural habitat: *X*^2^_1,31_=8.20, *P*=0.004). Lines indicate back-transformed model predictions for dominant (dashed) and all other bee species (solid). (**c**) The relation between the proportion of semi-natural habitat in agricultural landscapes and the bee species richness differs between dominant crop-visiting species and all other species (interaction type of bee and cover semi-natural habitat: *X*^2^_1,31_=7.84, *P*=0.005). Lines indicate back-transformed model predictions for dominant (dashed) and all other bee species (solid).

The dominant crop-visiting bee species dominate bee communities in agricultural landscapes generally, constituting 75.4±6.9% of individuals in these habitats in Europe and 59.2±10.5% in North America. This suggests that the species that are the dominant crop pollinators are the most widespread and abundant species in agricultural landscapes in general. Furthermore, the proportion of all bees on crops that belong to the dominant crop-visiting species was inversely related to the proportion of semi-natural habitats around study sites (Fig. 2a), and declined from ∼92% in landscapes almost completely devoid of semi-natural habitats to 40% in landscapes with half of the area covered by semi-natural habitats. This occurred because the pooled number and species richness of dominant crop-visiting bees were not related to semi-natural habitat cover, whereas the pooled number and species richness of all other bee species declined with decreasing cover of semi-natural habitat (Fig. 2b,c).

### Mitigating loss of dominant crop-visiting bee species

**Figure 3:**
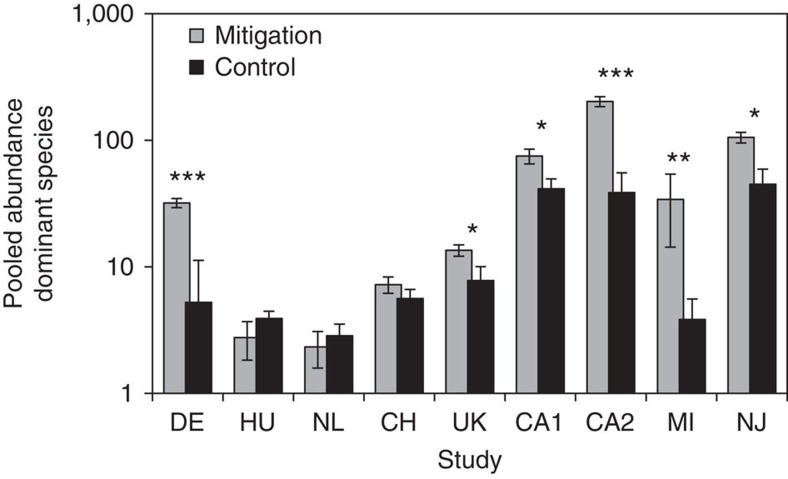
The effect of measures mitigating biodiversity loss on dominant crop-visiting bee species. Bars indicate mean pooled abundance (±s.e.) of dominant crop-visiting bee species on sites with management measures mitigating biodiversity loss compared with control sites in nine different studies. Abbreviations and test statistics: DE—Germany, *F*_1,40_=12.69, *P*<0.001; HU—Hungary, *F*_1,38_=1.13, *P*=0.295; NL—Netherlands, *F*_1,39_=0.36, *P*=0.553; CH—Switzerland, *F*_1,39_=1.29, *P*=0.263; UK—United Kingdom, *F*_1,39_=4.97, *P*=0.032; CA1—California study 1, *F*_1,37_=6.97, *P*=0.012; CA2—California study 2, *F*_1,9_=29.83, *P*<0.001; MI—Michigan, *F*_1,5.6_=15.10, *P*=0.009; NJ—New Jersey; *F*_1,10_=10.06, *P*=0.010. *P<0.05, **P<0.01, ***P<0.001.

To test whether dominant crop-visiting species can easily be enhanced (hypothesis 3), we compared their abundance on sites with biodiversity-enhancing management with that in ‘background' agricultural habitats (as defined above). Across all studies, biodiversity management raised the abundance of dominant crop-visiting bees by a factor of 3.2. Organic farming, planting wildflowers and establishing grass margin strips significantly enhanced dominant crop-visiting bees in arable landscapes (Fig. 3). On grasslands, restricting the use of agro-chemicals and delaying the annual onset of agricultural activities (Hungary, Switzerland and the Netherlands; Fig. 3) did not result in increased densities of dominant crop pollinators.

## Discussion

Here we show that wild bee pollinators provide important pollination services to crops around the globe (Fig. 1a), with the economic value of this ecosystem service being on par with that provided by managed honey bees. Knowledge of the economic contribution of wild pollinators to farm income points out the potential for win–win situations, as it allows for the identification of cost-effective measures that raise both crop yields and promote wild pollinator populations^25^. However, our results also clearly highlight the limitations of the ecosystem services argument for biodiversity conservation, because we found that only a small minority of common bee species provides most of the crop pollination services.

Our data sets supported all three of our hypotheses about the disconnect between the ecosystem services approach to conservation and the protection of biodiversity at large. First, few species are needed to provide ecosystem services, with almost 80% of the crop pollination provided by only 2% of bee species. Second, the species currently contributing most to pollination service delivery are generally regionally common species, whereas threatened species contribute little, particularly in the most agriculturally productive areas. Thus, a strictly ecosystem-service-based approach to conservation would not necessitate the conservation of threatened species. Third, the most important ecosystem-service-providing species are relatively robust to agricultural intensification, and furthermore can be readily enhanced in those systems by simple management actions. This suggests that the rarer species, which are already absent from such systems, would benefit less from ecosystem-service-based actions than they would from traditional biodiversity conservation that targets threatened species in the areas where they are found.

The first two points have been raised before in opinion and perspective papers as arguments for why the usefulness of ecosystem service provision as an argument to conserve biodiversity may be limited^18^,^19^,^20^. The contribution of this study is that we bring large data sets to this question for the first time. Specifically, for hundreds of bee species, we quantify both the economic value of the ecosystem services they provide as well as their conservation status. Such empirical testing in real-world landscapes is essential, given that, at present, the conclusion that ecosystem functioning strongly benefits from increased biodiversity rests primarily on data from small-scale experiments^12^. At the same time, the ecosystem services argument for conservation is gaining considerable traction as a dominant paradigm in real-world conservation^6^,^7^,^8^.

At first sight, our findings contrast with results of earlier studies, several of which were part of this study^26^,^27^,^28^,^29^, that demonstrated the benefits to crop production of pollinator biodiversity. The observed positive relations between pollinator species richness and seed or fruit set indicate that, at the plant or field scale, more diverse pollinator communities generally provide better pollination services (summarized in ref. 30). Our finding that relatively few species dominate pollination service delivery is largely the result of the larger spatial scale and the consideration of species identity in this study. Accounting for the identity of species shows that pollinator communities in different farm fields across large areas basically consist of variations of the same core set of species that prefer to forage on crops and that are augmented with the occasional new species. So while there is little doubt that a reduction in the local diversity of crop-visiting bee species may have negative consequences for the pollination services they deliver^26^,^27^, here we show that even the cumulative number of species across species-poor and species-rich fields represents only a small proportion of all bees and are dominated by an even smaller subset of species that occur on most fields (Fig. 1b).

One benefit of biodiversity to ecosystem services is that it may provide insurance effects that stabilize services over time or space^31^. Our results are in line with this because for most bee species large contributions to pollination were limited to specific years, crops and/or sites (Fig. 1a). It could therefore be argued that in order to maintain stable pollination services, one would need to conserve a much wider set of bee species than those that are currently numerous on crops. Species that are now rarely observed may, after all, become important in the future. While this may be true, this line of reasoning only applies to bee species that can actually use crop plants for forage. Bee species, even generalists, have distinct preferences for host plants^32^ and may be incapable of raising offspring on resources from non-preferred plants such as agricultural crops (cf. ref. 33). Species preferring non-crop plant families show more negative population trends than species specializing on members of crop plant families^34^,^35^, thereby confirming that many bee species fail to make use of this abundant resource supply. Thus, many of the bee species that are currently absent from crop flowers are unlikely to be important for spatial or temporal insurance effects of pollinator biodiversity on crop pollination, simply because they will not utilize crops even if conditions change.

Many previous studies have found that species richness of bee communities in agricultural landscapes declines with decreasing proportion of semi-natural habitats^36^,^37^. Our findings present a novel and more nuanced interpretation: while most bee species decline in abundance with expansion of agriculture, the species currently providing most of the pollination services to crops persist (Fig. 2b). Previous studies on plants have likewise demonstrated that with increasing land use intensity subdominant species are the first to decline, whereas dominant species are little affected^38^,^39^. Whether bee communities consisting of only the dominant pollinators are capable of providing sufficient pollination is unclear, but this pattern suggests that land use change will affect crop pollination less than it affects biodiversity^12^.

Measures to mitigate loss of pollination services are most cost effective in relatively intensively farmed landscapes because here measures have the highest impact^40^, ecosystem service delivery is likely to be reduced owing to the intensive farming practices, and returns on investments are greater owing to higher yields in intensively farmed areas^39^. Our results show that pollinator habitat creation in intensively farmed landscapes can successfully enhance the dominant crop-visiting bee species (Fig. 3), but are unlikely to benefit threatened species because of lack of source populations^17^. Species are classified as threatened when their numbers have experienced significant declines or their geographical distributions have contracted. Agricultural intensification is an important driver of species decline^1^. It is therefore perhaps not surprising that, in agricultural landscapes, threatened species contribute little to ecosystem service delivery, and benefit little from general conservation measures^17^. However, in the past, many of the species that are now threatened occurred widespread and contributed to pollination services on more extensively managed farmland^41^. Threatened species may also still dominate bee communities in restricted parts of their former distributional range^42^. Effective conservation measures for threatened species should therefore be targeted towards these bee species and their habitats, and not the crops to be pollinated^39^,^43^.

Highlighting the economic benefits people might obtain from biodiversity can be an effective instrument to motivate people or institutions to support biodiversity conservation. However, too much focus on the services delivered by pollinators may lead to adoption of practices that will not benefit species that could potentially contribute under changing agricultural conditions nor species that will never contribute to crop pollination. Benefits of biodiversity should therefore not be used as the sole rationale for biodiversity conservation as, for example, is currently done in the new strategy of the Convention on Biological Diversity^7^ and in the EU biodiversity strategy to 2020 (ref. 8). Moral arguments remain pivotal to supporting conservation of the larger portion of biodiversity including threatened species that currently contribute little to ecosystem service delivery. Such arguments are powerful and define many human actions, from taking care of the elderly to preserving historical buildings or art^44^. Ecologists and conservationists need to make these distinctions clear if we expect policy makers or land owners to defend species with no clearly defined economic value to humans.

## Methods

### Data sets to study crop visitation by bees

Our data sets record the relative visitation rate of bees to crop flowers, which is a good proxy for the relative contribution to pollination service delivery (see next section). We used data from 90 studies and 1,394 crop fields around the world that used standardized protocols to examine the abundance and identity of wild bees visiting flowers of 20 different crops that depend on bee pollinators for maximum yield (Supplementary Fig. 1 and Supplementary Table 1). We determined species abundance distributions of wild bee communities on insect-pollinated crops by pooling data within studies, that is, from fields sampled in the same year, region and crop species. We only included studies that directly observed individual bees on crop flowers, identified all individuals to species level and that were based on data from at least four fields that were 1 km or more apart. This yielded a total of 90 studies with an average of 15.7 fields per study that were on average 41.7 km apart.

### Flower visitation frequency as a proxy for crop pollination service delivery

Pollination is a function of both pollinator visitation frequency to flowers and per-visit pollen deposition (or efficiency)^45^. Because the differences in per-visit pollen deposition among species are generally outweighed by the differences in flower visitation among species^46^, visitation frequency is considered to be a good proxy for total pollination per species^47^. However, previous analyses of the suitability of visitation as proxy for pollination are mostly based on non-crop species (only 3 out of 22 species analysed by ref. 47 are crops, namely *Citrullus lanatus*, *Helianthus annuus* and *Phaseolus coccineus*). We therefore additionally analyse the relationship between visitation frequency (measured as the number of individual bees collected from crop flowers), per-visit pollen deposition (measured as the number of conspecific pollen grains deposited during a single visit^45^,^46^,^47^) and total pollination (calculated as the product of these two terms) using four of our best-resolved crop-pollinator data sets. The crops included are watermelon (5 years), tomato (2 years), cranberry (2 years) and blueberry (2 years), such that overall we analysed 11 crop-year combinations. Each annual data set was treated separately because different sites were studied in different years, and also because pollinator populations can fluctuate considerably among years. Each crop data set included extensive data on single-visit pollen deposition, a common metric used to assess per-interaction efficiency^46^ (watermelon 302 single-visit pollen deposition experiments conducted with virgin flowers, cranberry 176 experiments, blueberry 100 experiments and tomato 66 experiments; for methods details see refs 48, 49, 50). Because our data on per-visit pollen deposition were resolved only to the level of species groups, we combined our visitation data into the same groups to avoid biasing our analyses with respect to the variance contributed by the visitation as compared with the pollen deposition factors (see below). At least one known nectar robber (*Xylocopa virginica*) was included in several of our data sets. This would tend to increase the importance of per-visit deposition, and decrease the importance of visitation, in driving total pollination, which is a bias against the assumption tested here.

We calculated total pollination as visitation multiplied by per-visit pollen deposition, as is generally done in the literature^47^, and then examined the Pearson correlations between each of these three values. Values of Pearson's *r* between visitation and total pollination were high (mean=0.87; Supplementary Table 4). Although our methodology for estimating total pollination as the product of visitation and per-visit deposition makes such a correlation likely, it does not constrain it to be the case. The same expectation applies to per-visit deposition, which was not strongly correlated with total pollination (mean *r*=0.11; Supplementary Table 4). Furthermore, visitation and per-visit deposition were not correlated (Supplementary Table 4). Interestingly, our crop data sets reveal the same mechanism found by ref. 47 using data sets on predominantly native plant species: the high correlation arises because visitation has a much larger variance than does per-visit deposition; thus, visitation drives the variance in total pollination (Supplementary Table 4). In conclusion, there is strong empirical evidence that visitation is a good proxy for pollination in our data sets.

### Determining species abundance distributions

To be able to determine species abundance distributions, we only used studies that identified all bee individuals to species level. However, this was not possible for a small number of species complexes. On mainland Europe, *Bombus terrestris* and *B. lucorum* workers and queens are extremely difficult to separate without careful microscopic examination or molecular techniques, and so are nearly always grouped together in field studies^51^. In this study, they were therefore considered as a single taxon. In the eastern United States, *Ceratina calcarata, C. dupla* and *C. mikmaqi* were grouped for similar reasons, as were *Lasioglossum leucocomum* and *L. pilosum*. The western honey bee (*Apis mellifera*), was only considered to be non-managed in South Africa because here the species is native and wild populations still exist (although managed honey bees are also used to enhance pollination of some crops, such as apples). In Indonesia, the Asian honey bee (*A. cerana*) is occasionally kept by local people and so was considered to be a managed pollinator. In all other countries, honey bees were considered to be managed pollinators and therefore irrelevant for ecosystem service provisioning. However, honey bee abundance was incorporated in the calculations of the contribution of bees to crop production value. On average, western honey bees had similar flower visitation rates as wild bees (proportional contribution: 0.51±s.e. 0.036), although this varied among crops (Supplementary Table 1). Across all studies, species abundance distributions were based on 754 individuals.

### The economic contribution of bees to crop production

For 53 studies, the data allowed us to calculate the economic contribution of wild bees to crop production using the production value method^22^. The financial contribution of individual pollinators to crop production was estimated using national Food and Agriculture Organization of the United Nations statistics for each crop^52^, year and country combination, and the production value method^53^: *V*_Δpollination_=*P*·*Y*·*D*·*ρ*. Here *V*_Δpollination_ is the value of pollination ($ ha^−1^), *P* is the price ($ tonne^−1^), *Y* is the yield (tonne ha^-1^), *D* is the proportional reduction in crop yield without pollination^54^ and *ρ* is the proportion of the visits to crop flowers made by a particular bee species (including honey bees).

### Identifying dominant crop-visiting bee species

Bee species were characterized as being dominant within a study when their relative abundance on crop flowers was 5% or higher. This threshold corresponds to the cumulative set of species that collectively provide 80% of the crop flower visits (Supplementary Fig. 2). Sensitivity analysis on this choice of threshold showed that results were robust to the choice of threshold so long as the definition of ‘dominant' did not fall below including species that contributed only 2% of total crop flower visits (Supplementary Fig. 3). Furthermore, our results regarding the dominant crop-visiting species were robust to various study designs and methodological differences among studies, including the spatial extent of sampling and sampling effort (Supplementary Fig. 4). Last, as is often the case for studies of bees for which identification keys do not exist for many parts of the world, there were some unidentified specimens in our studies. These difficult-to-identify taxa were generally rare, however (when pooled, still <5% of the specimens in a given data set), and thus would have minimal impact on our main analyses.

### Crop-visiting bee species relative to regional species pool

Conservation policy objectives are often formulated at national or even continental levels. We therefore also explored how the number of bee species encountered in our studies compared with the total number of unique bee species existing in the political territories in which the studies were performed (that is, the regional species pool). We used a database compiled from published and unpublished sources by J.S.A. of all described bee species currently known to exist in each country, state or province (that is, at the lowest territorial level for which such lists could be obtained). We obtained these data for the German federal states of Hessen^55^, Lower Saxony^56^ and Bavaria^57^, and for the European countries of France, Great Britain, Hungary, Israel, Italy, Netherlands and Sweden (from ref. 58). In North America, species lists were obtained from ref. 58, for the US states California (CA), Massachusetts, New Jersey (NJ), New York, Pennsylvania and Virginia, and the Canadian province of British Columbia. Elsewhere in the world, species lists were used from ref. 58 for Chiapas (Mexico), Costa Rica, Minas Gerais (Brazil), New Zealand, South Africa and Sulawesi (Indonesia). We subsequently calculated straight-forward sample-based species accumulation curves using EstimateS software^59^, treating each territorial species list as a sample. Because each species list is not an ecological sample but is based on collections, revisions, faunal surveys and national inventories, we refrained from calculating a true species richness estimator.

To examine what proportion of the regional bee species pool visited crop flowers, and what proportion of them was dominant in at least one study, we similarly generated species accumulation curves for (dominant) crop-visiting bee species. Using the full data set of all observed bee species on crop flowers in our data set, we computed the nonparametric, asymptotic true species richness estimator Chao1 with log-linear 95% confidence intervals^60^, which corrects for unseen species based on the number of species in each study that were observed once (singletons) or twice (doubletons). For dominant species, which included no singletons or doubletons, and further are unlikely to include missing species, we calculated straight-forward species accumulation curves.

### The contribution of threatened species to crop visitation

To examine what proportion of the bee communities observed on crops had a recognized threat status, we used Red Data Books. Red Data Books were only available for four of the countries from which we had data of crop-visiting bee species: Germany^61^, Netherlands^62^, Sweden^63^ and United Kingdom^64^. In total, 19 separate studies had been carried out in these countries for which we calculated the per study mean pooled proportion of individuals from threatened species.

### Data sets to study commonness and effects of conservation

To address the hypotheses that dominant crop-visiting bee species are generally common species and that these species can be easily enhanced by simple management actions, we used data from a number of European and North American studies examining the effects of measures to promote biodiversity in agricultural areas. These studies used paired designs and standardized protocols to compare bee community composition on sites with biodiversity-enhancing management with that on control sites (sites that were as similar as possible to the treatment sites but were not exposed to biodiversity management). Full details of the study locations and methodologies of the European studies collected in the EU-funded EASY project are given in refs 17, 65. In summary, these sites were sampled in Germany, Hungary, Switzerland, the Netherlands and the United Kingdom in 2003. In each country, three regions were selected with contrasting landscape structure with each region containing seven field pairs. Biodiversity-enhancing management involved delaying the first seasonal cut of grasslands, restricting agro-chemical usage, and/or restricting cattle stocking rates (Hungary, Switzerland and The Netherlands), organic arable farming (Germany) and establishing 6-m-wide grass field margin strips along arable fields (the United Kingdom); all interventions were in the framework of existing agri-environment schemes. In each field, all samples were taken along two 95-m-long transects: one along the field edge and another, parallel to the first one, 50 m from the edge in the grassland interior. We sampled bees using sweep nets (60 sweeps per transect per round) and transect surveys (15 min sampling per transect per round) in the edge and interior of the fields three times (May, June and July) in 2003. For analyses, all data per field were pooled.

In the United States, unpublished 2012 data were used from two studies in CA, one in NJ and one in Michigan (MI). Biodiversity-enhancing management involved establishment of hedgerows of native perennial plants (study CA1) or establishment of wildflower plantings (studies CA2, NJ, MI). In contrast to the European studies, experimental sites in the United States were generally located adjacent to agricultural fields on pre-existing field edges or old fields. For the CA1 study, 20 field edges were selected containing native plant restorations (all at least 5 years old), which were paired with 20 non-restored control sites. Restorations were ∼350 m long and 3–6 m wide and contained a mix of native perennial shrubs and trees^24^. Control sites were selected to roughly match conditions surrounding paired restoration sites; for each restoration site, a control site was selected adjacent to the same crop type (row crop, orchard, pasture or vineyard) within the same landscape context (that is, within 1–3 km of the restoration site), but at least 1 km from all other study sites. Control sites were generally weedy field edges and they reflected a variety of unmanaged crop field edges found in the region. Bee communities were sampled at each restoration and control site four times (except one pair of sites sampled only three times). Bees were netted along a 350-m transect for 1 h, stopping the timer while handling specimens. All native bees were collected and identified in the laboratory. The other three studies (CA2, NJ and MI) used the same general approach; each had six site pairs consisting of a wildflower plot established at least 2 years before sampling, using diverse (at least 10 species) mixes of native wildflowers that provided resources for bees throughout the growing season, paired with a control plot that was unrestored. Sampling sites within each pair were separated by 100–800 m. In NJ, four 40 m transects were established within each plot and sampled once in the morning and once in the afternoon, for 10 min each (net sampling time). In MI and CA2, eight 23-m-long transects were established in each plot and were sampled once in the morning and once in the afternoon for 5 min. All bees visiting flowers within 1 m of the transect were collected. In all three studies, each site was sampled four times throughout the summer. Again, for analyses, all data per site were pooled.

### Analysing commonness in relation to semi-natural habitat

To examine whether dominant crop-visiting bee species are common species in agricultural landscapes, generally (hypothesis 2) only data from the control sites were used because they were situated in agricultural habitats such as arable fields (but not flowering, bee-pollinated crops), grasslands, old fields and hedgerows. The proportion of the bee communities consisting of individuals from bee species dominating crop vistitation rates (Supplementary Table 3) were then calculated. The units of analysis were averages of multiple fields, as sample size per site was too low to yield reliable estimates of the relative contribution of dominant species to the bee community. In Europe, averages per region within each country (*n*=7) were used, whereas in the United States the average per study was used. For the studies MI, NJ and CA2, sample size was six, whereas for CA1 sample size was nine, since land cover data (see below) for all 20 site pairs were not available. To explain differences in the proportional contribution of dominant species between studies, this variable was tested against a number of variables known to affect bee species community composition: the percentage of semi-natural habitat in the vicinity of sampling sites, latitude and continent^26^. The percentage of semi-natural habitat (for example, extensive grasslands, forests, heathlands and wetlands) was calculated in a radius of 1,000 m around each site, an approximate mean range at which different species groups of bees have been shown to respond to semi-natural habitat in studies on different continents^48^,^66^. For the European sites, we used CORINE Land Cover 2006 data sets^67^ (all land use classes with codes starting with 3 or 4) which, although less accurate than national data sets, provide spatially consistent land cover classifications across all countries. In NJ, land cover data sets provided by the State Department of Environmental Protection were used (http://www.nj.gov/dep/gis/lulc07cshp.html). In MI, land cover was manually digitized from 2012 National Agriculture Imagery Program orthoimagery at the 1:2,000 scale (United States Department of Agriculture Geospatial Data Gateway, http://datagateway.nrcs.usda.gov/). The other two US studies used the National Agricultural Statistics Service crop data file (http://nassgeodata.gmu.edu/CropScape/).

We used standard multiple linear regression models to relate the proportion of individuals from dominant crop-visiting species in bee communities to the proportion of semi-natural habitat, thereby correcting for latitude and continent. Plotting residuals versus fitted values confirmed that model assumptions were met satisfactorily. The often used arcsine transformation of proportional data or binomial regression increased heteroscedasticity, and we therefore present the results of untransformed data. To subsequently explain the patterns in the proportional data, we calculated standardized abundances of dominant crop-visiting bees and, separately, for all other bees for each of the European study regions by dividing the per region bee abundance by the mean abundance across all 15 regions. Since the study in each region had used exactly the same survey protocol, a standardized bee abundance >1 indicates above-average bee abundance compared with the cross-study mean, and a value <1 indicates a below-average bee abundance. We similarly calculated standardized abundances of dominant crop-visiting bees and, separately, all other bees for the three US studies that used the same survey protocol (study CA1 used a different survey protocol and was excluded from this particular analysis). The same approach was used to calculate per study standardized species richness. This allowed us to use the European and US data sets in a joint analysis. We used log-linear models assuming a Poisson distribution with standardized abundance or species richness as response variables, and the proportion semi-natural habitat, bee type (dominant crop-visiting bees versus all other bees) and their interaction as main explanatory variables of interest. A significant interaction would indicate that dominant crop-visiting bees and all other bees are differently related to semi-natural habitat. Latitude was again included as a correcting variable. Continent was not included because we had standardized the response variables between the studies on each continent.

### Analysing effects of measures mitigating biodiversity loss

We used site-level count data as the statistical unit and used generalized linear mixed models assuming Poisson error distribution and using a log-link function^68^. The initial models used treatment pair as a random term and study, mitigation measure (yes and no) and their interaction as fixed terms. This revealed a significant interaction between the effects of mitigation measures and study (*F*_8,267_=3.94, *P*<0.001). We therefore chose to perform separate analyses for each study with treatment pair as a random factor and mitigation measure as a fixed factor. We chose not to correct for multiple testing, as correction reduces type I error, but tends to inflate type II error^69^. Instead, we critically interpret statistical outcomes of analyses comparing treatment means. Model outcomes were checked by plotting residuals versus fitted values, confirming that assumptions were met satisfactorily.

All models were fitted using standard facilities in Genstat^70^.

## Additional information

**How to cite this article:** Kleijn, D. *et al.* Delivery of crop pollination services is an insufficient argument for wild pollinator conservation. *Nat. Commun.* 6:7414 doi: 10.1038/ncomms8414 (2015).

## Supplementary Material

Supplementary Information Supplementary Figures 1-4, Supplementary Table 1-4, and Supplementary References.
