# Corrigendum: Delivery of crop pollination services is an insufficient argument for wild pollinator conservation

**DOI:** 10.1038/ncomms10841

**Published:** 2016-02-18

**Authors:** David Kleijn, Rachael Winfree, Ignasi Bartomeus, Luísa G Carvalheiro, Mickaël Henry, Rufus Isaacs, Alexandra-Maria Klein, Claire Kremen, Leithen K. M'Gonigle, Romina Rader, Taylor H. Ricketts, Neal M. Williams, Nancy Lee Adamson, John S. Ascher, András Báldi, Péter Batáry, Faye Benjamin, Jacobus C. Biesmeijer, Eleanor J. Blitzer, Riccardo Bommarco, Mariëtte R. Brand, Vincent Bretagnolle, Lindsey Button, Daniel P. Cariveau, Rémy Chifflet, Jonathan F. Colville, Bryan N. Danforth, Elizabeth Elle, Michael P. D. Garratt, Felix Herzog, Andrea Holzschuh, Brad G. Howlett, Frank Jauker, Shalene Jha, Eva Knop, Kristin M. Krewenka, Violette Le Féon, Yael Mandelik, Emily A. May, Mia G. Park, Gideon Pisanty, Menno Reemer, Verena Riedinger, Orianne Rollin, Maj Rundlöf, Hillary S. Sardiñas, Jeroen Scheper, Amber R. Sciligo, Henrik G. Smith, Ingolf Steffan-Dewenter, Robbin Thorp, Teja Tscharntke, Jort Verhulst, Blandina F. Viana, Bernard E. Vaissiére, Ruan Veldtman, Kimiora L. Ward, Catrin Westphal, Simon G. Potts

*Nature Communications* 6: Article number: 741410.1038/ncomms8414 (2015); Published: 06162015; Updated: 02182016.

The authors inadvertently omitted Kimiora L. Ward, who managed and contributed data, from the author list. This has now been corrected in both the PDF and HTML versions of the Article.

