# Supplementary material for: Delivery of crop pollination services is an insufficient argument for wild pollinator conservation
Source: Nat Commun. 2015 Jun 16;6:7414. doi: 10.1038/ncomms8414 (PMC4490361; doi:10.1038/ncomms8414)
Supplement: Supplementary Information — Supplementary Figures 1-4, Supplementary Table 1-4, and Supplementary References. [file ncomms8414-s1.pdf]

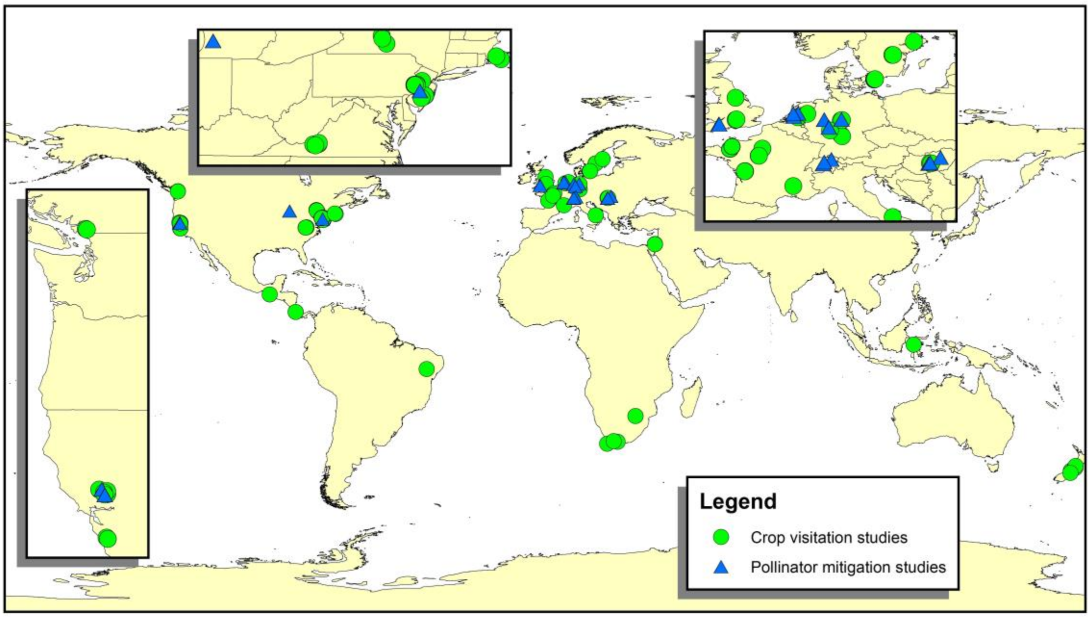

Supplementary Figure 1 | An overview of the study site locations. Note that some of the symbols are overlapping where a study had temporal (yearly) replicates. Further details of studies are given in Supplementary Table 1.

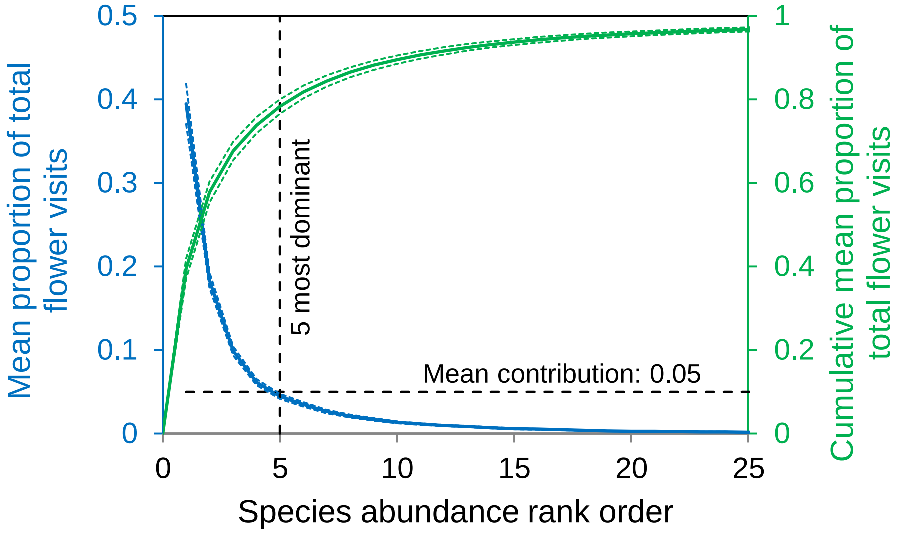

Supplementary Figure 2 | The relationships between the species abundance rank order and the mean (cumulative) proportional contribution of species to the total crop-visitation by wild bee species. Depicted are means (±s.e. indicated by dashed lines) of 90 studies.

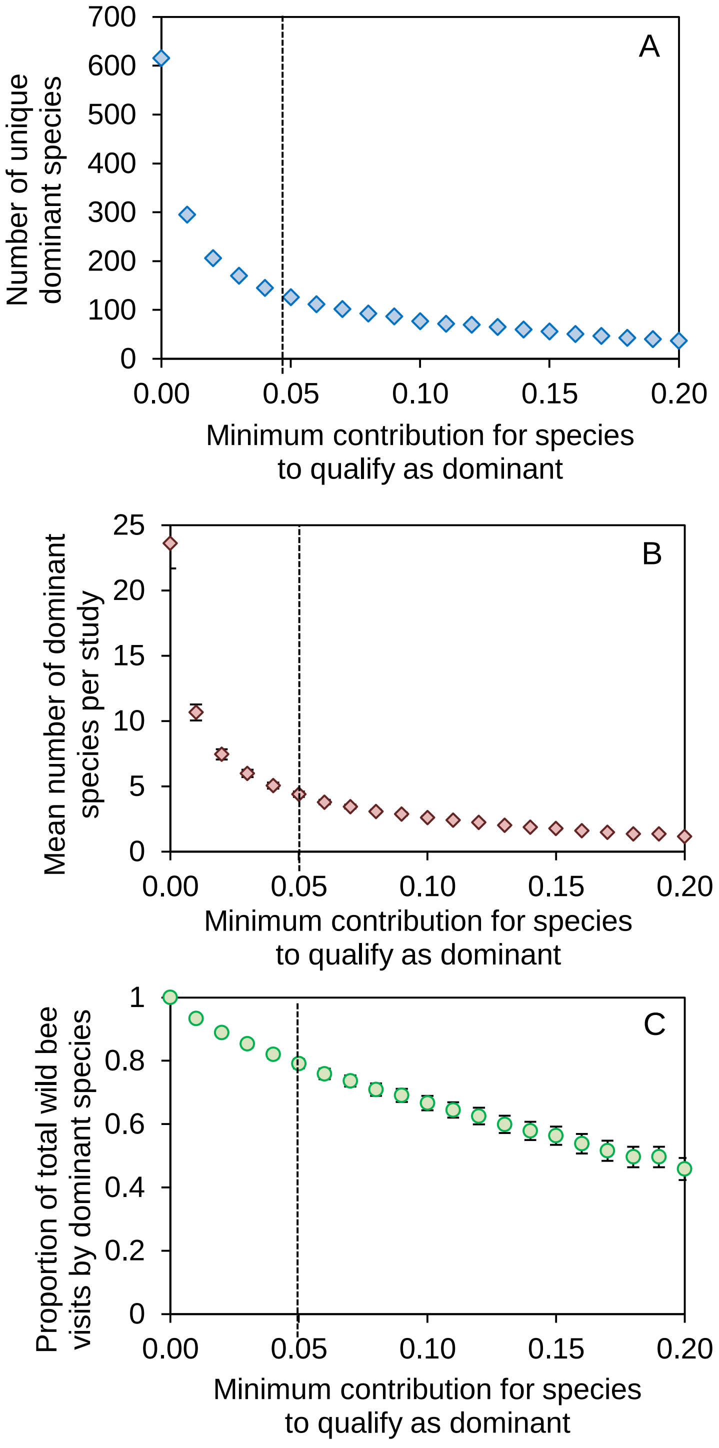

Supplementary Figure 3 | The relationship between threshold level for identifying dominant crop-visiting bee species and the proportion of the total wild bee visits made by dominant bee species. Dominance patterns are relatively robust to changes in the dominance criterion. Changing the threshold value from the 5% value used in this study to characterise dominant species (vertical dashed line), results in only modest changes in: **a,** the total number of unique dominant bee species across all studies; **b,** the mean (± se, n = 90) number of dominant species per study; and, **c,** the mean (± se, n = 90) contribution by dominant species to the total bee flower visits. Only when the dominance criterion drops below 2%, do changes become marked.

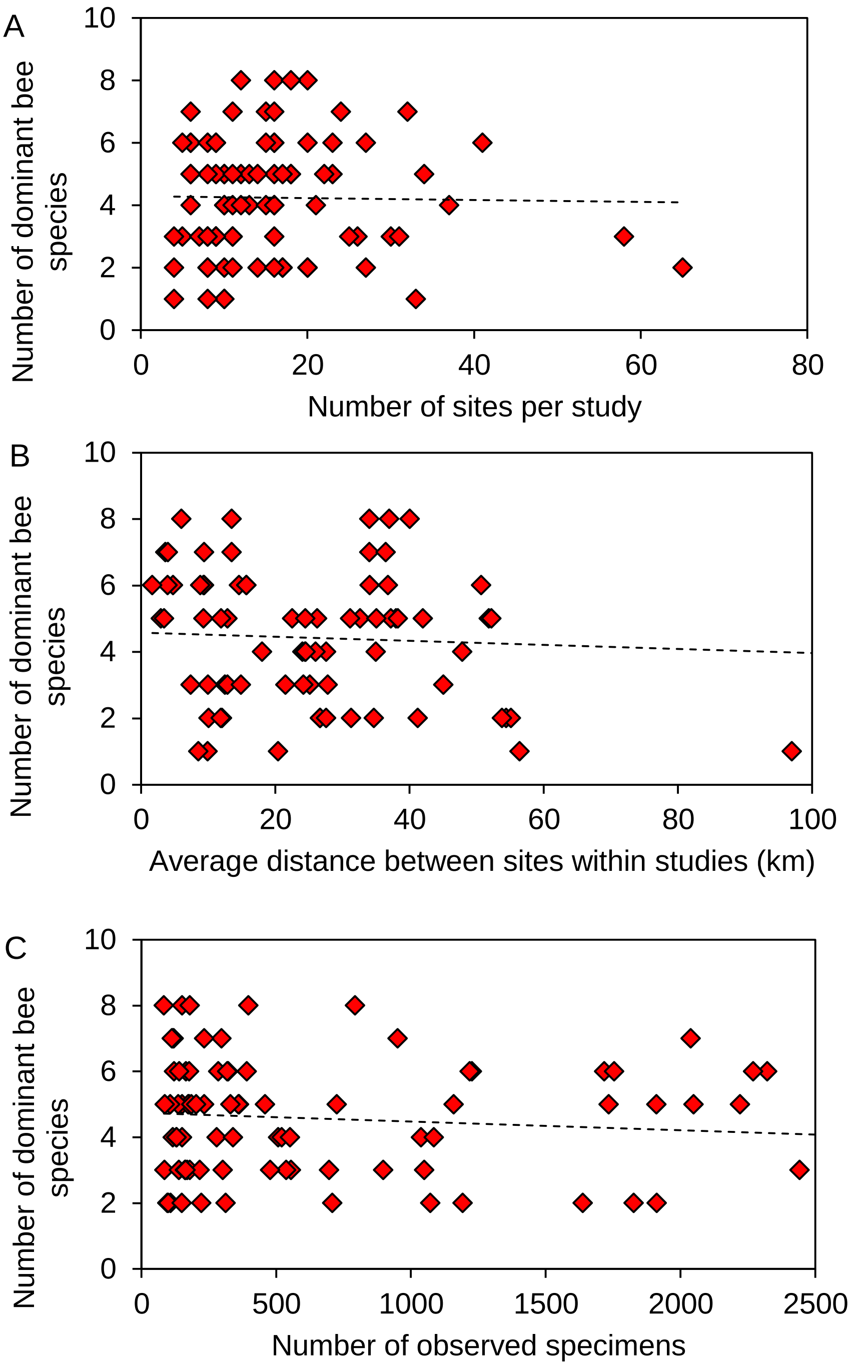

Supplementary Figure 4 | The relationships between the number of identified dominant bee species visiting crop flowers and a number of key characteristics of the studies. a, The number of dominant species was not related to the number of sites sampled in a study (simple regression analysis; $F_{1,83} = 0.03$, $P = 0.875$) and was constant at about ~4.2 species across the entire range of 4-65 sites sampled per study. **b,** The number of dominant species was not related to the average distance between the sites that had been sampled in each study ($F_{1,80} = 2.77$, $P = 0.100$). Within studies, sites were at least 1 km apart. Studies with average between site distances larger than 100 km (21-23, 76-79, 86; see Extended Data Table 1) are not shown for clarity. **c,** The number of dominant species was not related to the number of observed individuals ($F_{1,83} = 2.13$, $P = 0.148$). South African studies were excluded from this

particular analysis as in South Africa it is impossible to distinguish wild from managed honey bees. Studies with more than 3,000 individuals are not shown (studies 9, 23; Supplementary Table 1). Dominant species contributed more than 5% to the total number of individuals of the bee community on crop flowers in each study. For all three analyses, regression on the full dataset revealed heteroscedasticity. This variability was caused by a small number of outliers (i.e. studies with very high between site distances). Transformations of the response variable, or analyses assuming binomial or Poisson error distributions, did not produce homoscedastic datasets. Analyses without these studies produced constant error variance. The results of these analyses were similar to those of analyses using the full dataset, and so in this figure, we therefore present the results using the full dataset. For illustrative purposes only, the non-significant relationships are depicted by means of a dashed trend-line based on these analyses.

Supplementary Table 1 | A summary of the studies providing the data on bee species visiting flowers of insect pollinated crops. Unpublished datasets are identified by the name of the first author. Yield and price statistics based on FAO data[25]. N.a.: not available.

Study no[reference] Crop; Location; year; yield (tonnes.ha^{-1}); price (\$.tonne^{-1}); no. sites; no. wild bees; ratio wild/honey bee

1[1] Red Clover (*Trifolium pratense*); Sweden, Östergötland; 2008; n.a.; n.a.; 14; 1637; 0.80

2[1] Red Clover (*Trifolium pratense*); Sweden, Skåne; 2009; n.a.; n.a.; 20; 1826; 0.62

3[1] Red Clover (*Trifolium pratense*); Sweden, Östergötland; 2009; n.a.; n.a.; 17; 1912; 0.74

4[1] Red Clover (*Trifolium pratense*); Sweden, Skåne; 2010; n.a.; n.a.; 11; 555; 0.53

5[1] Red Clover (*Trifolium pratense*); Sweden, Östergötland; 2010; n.a.; n.a.; 17; 1071; 0.53

Method: Bee abundances were recorded in arable fields of flowering red clover cultivated for seed production. In 2008, 2009 and 2010, flower visiting insects were collected along 1 m wide and 50 m long transects in the flowering red clover seed fields. In 2008, surveys in each field were based on four transects located 4 and 12 m from the field edge. In 2009 and 2010, surveys in each field were based on two transects located 8 and 100 m from the field edge (or for smaller fields in the field centre). Each site was visited two times in 2008 and three to five times in 2009 and 2010. Sampling was conducted only on days with warm, sunny and calm weather between 25 June and 29 July, 2008, 26 June and 20 August, 2009, and 5 July and 10 August, 2010. The collected bees were identified to species level. Of the bumble bee (*Bombus*) individuals, 2.8% were only identified to genus level because field workers did not catch them. As *Bombus* is a well-known genus with most individuals identified to species level, for these studies the unidentified specimens were assigned to species based on the proportions of actual species level identifications within each study.

Study no[reference] Crop; Location; year; yield (tonnes.ha^{-1}); price (\$.tonne^{-1}); no. sites; no. wild bees; ratio wild/honey bee

6[2] Oil Seed Rape (*Brassica napus*); Sweden, Uppsala; 2005; 2.41; 271.5; 10; 96; 0.06

Method: Bees were surveyed in ten oilseed rape fields. In each field, surveys were conducted in a 150 m long and 4 m wide transect line at the center of the field or, for large fields, between the center of the field and one of its margins. Flower visiting bees were sampled with an aerial net for 30 minutes identifying specimens to species level. In each field, transects were monitored four times during the main flowering period from 27 June to 21 July in 2005, between 9.00 and 17.00, and only on days with temperature ≥15°C, no precipitation, dry vegetation, and low wind speeds (<40 km.h^{-1}). Of the bumble bee (*Bombus*) individuals, 26% were only identified to genus level because field workers did not catch them. As *Bombus* is a well-known genus with most individuals identified to species level the unidentified specimens were assigned to species based on the proportions of actual species level identifications within each study.

Study no[reference] Crop; Location; year; yield (tonnes.ha^{-1}); price (\$.tonne^{-1}); no. sites; no. wild bees; ratio wild/honey bee

7[3] Oil Seed Rape (*Brassica napus*); Germany, Lower Saxony; 2007; 3.44; 392.0; 34; 362; 0.25

Method: In 2007 bee densities were assessed on 34 flowering oilseed rape fields in Germany. Bees were recorded along 100 m transects with 1 m width in the field centre and at the field edge for 15 min per transect on two occasions during oilseed rape flowering in April and May. The edge transect was located 1 m into the oilseed rape field along the field edge; the centre transect started 10 m from the field edge and followed a lane toward the field centre. Sites were sampled between 10.00 and 17.00 h at temperatures above 15 °C on days with no rain, low or non-existent cloud cover and low wind speeds. All bees that could not be identified in the field were collected for subsequent identification in the laboratory.

Study no[reference] Crop; Location; year; yield (tonnes.ha^{-1}); price (\$.tonne^{-1}); no. sites; no. wild bees; ratio wild/honey bee

8[V. Riedinger] Oil Seed Rape (*Brassica napus*); Germany, Bavaria; 2011; 2.91; 619.4; 16; 150; 0.29

Method: In 2011 bee densities were assessed on 16 flowering oilseed rape fields in Germany. In each field, bees were surveyed twice between 18 April and 10 May, 2011, in two 150 m long and 1 m wide transects, one located along the edge of the field and the other in the centre of the field. In each

survey, each transect was surveyed for 15 minutes by slowly walking its length. Surveys were conducted between 09:00 and 18:00 at temperatures above 15 °C on days with no rain, low or non-existent cloud cover and low wind speeds. Honey bees and bumble bees were identified to species in the field, all other bees were collected for identification in the lab.

Study no[reference] Crop; Location; year; yield (tonnes.ha^{-1}); price (\$.tonne^{-1}); no. sites; no. wild bees; ratio wild/honey bee

9[4] Sunflower (*Helianthus annuus*); Germany, Bavaria; 2011; 1.98; n.a.; 16; 7747; 0.39

Method: In 2011 bee densities were assessed on 16 flowering sunflower fields. In each field, bees were surveyed between 4 July and 6 August, 2011, in two 150 m long and 1 m wide transects; one located along the edge of the field and the other in the centre of the field. Surveys were conducted between 09:00 and 18:00 at temperatures above 16 °C on days with no rain, low or non-existent cloud cover and low wind speeds. On two fields, edge and centre transects were surveyed four times. On three more fields, edge transects were surveyed three times but center transects only two times. All other fields were surveyed completely three times, each time for 15 minutes, while slowly walking along the transect and recording only pollinators on sunflower heads. Honey bees and bumble bees were identified to species in the field, all other bees were collected for identification in the lab.

Study no[reference] Crop; Location; year; yield (tonnes.ha^{-1}); price (\$.tonne^{-1}); no. sites; no. wild bees; ratio wild/honey bee

10[5] Oil Seed Rape (*Brassica napus*); Germany, Hesse; 2006; 3.73; 292.6; 23; 177; 0.54

Methods: In June and July, 2006 bees were surveyed in 23 oilseed rape fields. Bees were surveyed in up to three sampling points within each field (depending on the field size). Each survey lasted ten minutes and took place between 10:00 and 17:00 on sunny days with little wind. Each sampling point was surveyed up to three times, depending on flowering phenology. Surveys were carried out by two experienced ecologists. Bees were either identified in the field or collected for identification in the laboratory.

Study no[reference] Crop; Location; year; yield (tonnes.ha^{-1}); price (\$.tonne^{-1}); no. sites; no. wild bees; ratio wild/honey bee

11[6] Strawberry (*Fragaria x ananassa*); Germany, Lower Saxony; 2005; 10.90; 3351.1; 10; 507; 0.16

Methods: Bees were surveyed using standardized transect walks in 10 strawberry fields. Surveys were carried out from 27 April until 16 June, 2005 under good weather conditions with temperatures at least 15°C, no precipitation and a wind speed below 40 km.h^{-1}. Bees were surveyed for 30 min in a 150 m transect line identifying visiting insects at species level and catching unidentified species within a 4 m wide corridor. Each field was surveyed four times during the main flowering period of the crop. Collected specimens were pinned, labeled, and subsequently identified to species.

Study no[reference] Crop; Location; year; yield (tonnes.ha^{-1}); price (\$.tonne^{-1}); no. sites; no. wild bees; ratio wild/honey bee

12[7] Field Bean (*Vicia faba*); UK, Reading; 2005; 3.83; 149.5; 10; 1037; 0.86

Methods: Bees were surveyed using standardized transect walks in 10 field bean fields. Surveys were carried out between 09:00 and 17:00 from May to August, 2005 under good weather conditions with temperatures at least 15°C, no precipitation and a wind speed below 40 km.h^{-1}. Bees were surveyed for 30 min in a 150 m transect line identifying visiting insects at species level and catching unidentified species within a 4 m wide corridor. Each field was surveyed four times during the main flowering period of the crop. Collected specimens were pinned, labeled, and subsequently identified to species. 62.5% of the bumble bee (*Bombus*) individuals were only identified to genus level because field workers did not catch them. For these studies, as *Bombus* is a well-known genus with most individuals identified to species level, the unidentified specimens were assigned to species based on the proportions of actual species level identifications within each study.

Study no[reference] Crop; Location; year; yield (tonnes.ha^{-1}); price (\$.tonne^{-1}); no. sites; no. wild bees; ratio wild/honey bee

13[M. Garratt] Strawberry (*Fragaria x ananassa*), UK, West Yorkshire; 2011; 22.16; 3859.0; 8; 1192; 0.55

14[M. Garratt] Field Bean (*Vicia faba*); UK, Berkshire; 2011; 3.35; 274.0; 8; 537; 0.88

Methods: In 2011, bee visitation to field beans and strawberry fields was surveyed. For each crop, 8 fields were selected and 2*150m transects were walked between rows. For recording purposes, the transects were sub-divided into 3*50m transects, each of which was walked for 10 minutes. Any pollinators observed carrying out floral visits (legitimate only for beans) were recorded, if the pollinator could not be identified in the field, it was collected and identified in the laboratory. Three rounds of bean surveys were carried out at each field between the 10th and 25th of May and 3 rounds of strawberry surveys between the 18th of May and the 14th of June. All surveys were conducted only when temperatures exceeded 15 °C and when wind was light or non-existent. 12.8 % of the bumble bee (*Bombus*) individuals were only identified to genus level because field workers did not catch them. For these studies, as *Bombus* is a well-known genus with most individuals identified to species level, the unidentified specimens were assigned to species based on the proportions of actual species level identifications within each study.

Study no[reference] Crop; Location; year; yield (tonnes.ha^{-1}); price (\$.tonne^{-1}); no. sites; no. wild bees; ratio wild/honey bee

15[8] Apple (*Malus domestica*); Netherlands, Gelderland; 2010; 38.47; 576.0; 6; 165; 0.42

16[8] Apple (*Malus domestica*); Netherlands, Gelderland; 2011; 50.57; 745.2; 6; 297; 0.24

17[8] Pear (*Pyrus communis*); Netherlands, Gelderland; 2010; 34.27; 750.7; 6; 150; 0.85

18[8] Pear (*Pyrus communis*); Netherlands, Gelderland; 2011; 40.96; 678.4; 6; 285; 0.78

Methods: In 2010 and 2011 bee visitation rate on crop flowers was examined in 6 apple and 6 pear orchards. The same apple and pear orchards were used in both years. Each orchard was surveyed twice per year, once in the morning and once in the afternoon with at least three and at most seven days separating surveys. Surveying was conducted by four experienced entomologists between 23 April and 6 May, 2010 and between 8 and 20 April, 2011 under sunny conditions or scattered clouds. Temperatures ranged between 15 °C and 20 °C with calm wind to moderate breeze. Bees were surveyed using a single transect between two rows of trees along the length of each orchard with the transect subdivided into 25 m long plots (mean number of plots per orchard ± s.e.: 8.5±1.0 for apple and 9.7±0.5 for pear). In each plot all bees observed on apple or pear flowers during a 10 minute-period were identified to species. Easily recognizable species were generally identified in the field; all other species were collected and identified in the lab.

Study no[reference] Crop; Location; year; yield (tonnes.ha^{-1}); price (\$.tonne^{-1}); no. sites; no. wild bees; ratio wild/honey bee

19[J. Scheper] Oil Seed Rape (*Brassica napus*); Netherlands, Overijssel; 2011; 3.44; 501.5; 8; 312; 0.42

Methods: In 2011, bees were surveyed in 8 oilseed rape fields. One field was surveyed only once on 30 April, while all others were surveyed twice between 30 April and 30 May, once in the morning and once in the afternoon. In each field, bees were surveyed in two 1 x150 m transects located at the edge and in the interior of the field (>25 m from field edge). Transects were subdivided into three 1 m x 50 m plots. In each plot, bees visiting crop flowers were collected during a period of 5 minutes. Easily recognizable species were generally identified in the field; all other species were collected and identified in the lab. Surveys were carried out under dry weather conditions, with low to moderate wind speeds and temperatures above 15 °C.

Study no[reference] Crop; Location; year; yield (tonnes.ha^{-1}); price (\$.tonne^{-1}); no. sites; no. wild bees; ratio wild/honey bee

20[D. Kleijn] Leek (*Allium porrum*); Italy, Foggia; 2012; n.a.; n.a.; 10; 173; 0.74

Methods: In 2012, bees were surveyed at 10 leek fields in the province of Foggia, Italy. Each field was surveyed once between 19 and 21 June, 2012 under sunny weather conditions with temperatures above 20 °C and light or non-existent winds. In each field, bees were surveyed in a single 5 m long transect between two crop rows. During a period of 10 minutes (net observation time), all bees visiting leek umbels were noted. Easily recognizable species were generally identified in the field; all other species were collected and identified in the lab.

Study no[reference] Crop; Location; year; yield (tonnes.ha^{-1}); price (\$.tonne^{-1}); no. sites; no. wild bees; ratio wild/honey bee

21[9] Alfalfa (Medicago sativa); Hungary; 1954; n.a.; n.a.; 8; 2321; n.a.

22[9] Alfalfa (Medicago sativa); Hungary; 1955; n.a.; n.a.; 9; 2441; n.a.

23[9] Alfalfa (Medicago sativa); Hungary; 1956; n.a.; n.a.; 11; 3464; n.a.

Methods: Data were extracted from reference (*63*). Bees were surveyed on presumably 8 alfalfa fields in 8 different areas between 21 June and 22 August, 1954. Surveys were repeated from 11 July and 9 September, 1955 in the same 8 areas along with one additional area, presumably on 9 alfalfa fields. Finally, a total of 11 fields in the same 9 areas were surevey between 5 July and 21 August, 1956. The study areas were scattered across Hungary and in each area and year bees were surveyed during approximately 10 days. Total number of survey days per year were 67 in 1954 and 80 in 1955. In 1956, bees were surveyed for a total of 127 hours. Wild bees were collected with nets from alfalfa flowers. Individual surveys lasted 30 minutes and were conducted both in the morning and in the afternoon.

Study no[reference] Crop; Location; year; yield (tonnes.ha^{-1}); price (\$.tonne^{-1}); no. sites; no. wild bees; ratio wild/honey bee

24[10] Alfalfa (*Medicago sativa*); Hungary; 2004; n.a.; n.a.; >10; 1910; n.a.

25[10] Alfalfa (*Medicago sativa*); Hungary; 2005; n.a.; n.a.; >10; 950; n.a.

26[10] Alfalfa (*Medicago sativa*); Hungary; 2006; n.a.; n.a.; >10; 1158; n.a.

27[10] Alfalfa (*Medicago sativa*); Hungary; 2007; n.a.; n.a.; >10; 1717; n.a.

Methods: Data were extracted from reference (*64*). In the years 2004 through 2007, bees on alfalfa fields were surveyed throughout Hungary. Each year 120-160 surveys were made near 80 settlements in 5-19 different counties. This study represents a replication of the surveys conducted in studies 21-23.

Study no[reference] Crop; Location; year; yield (tonnes.ha^{-1}); price (\$.tonne^{-1}); no. sites; no. wild bees; ratio wild/honey bee

28[11] Sunflower (*Helianthus annuus*); France, Poitou-Charentes; 2010; 2.36; 563.9; 30; 85; 0.07

29[11] Alfalfa (*Medicago sativa*); France, Poitou-Charentes; 2010; n.a.; n.a.; 18; 136; 0.31

30[11] Oilseed rape (*Brassica napus*); France, Poitou-Charentes; 2011; 3.45; 569.7;58; 139; 0.19

31[11] Sunflower (*Helianthus annuus*); France, Poitou-Charentes; 2011; 2.54; 596.1; 65; 108; 0.02

32[11] Alfalfa (*Medicago sativa*); France, Poitou-Charentes; 2011; n.a.; n.a.; 41; 322; 0.11

Methods: In 2010 and 2011, bees were surveyed on three different crops in a 500 km^2 intensively farmed area in Western France (LTER "Zone Atelier Plaine & Val de Sèvre"). A total of 217 sites were surveyed, located in 30 grid cells (10 per year) randomly drawn without replacement from a 3×3 km grid covering the whole study area. Sampling took place during the flowering periods of oilseed rape (April 1 - 13 in 2011) and sunflower (July 16 - 23 in 2010 and June 28 - July 12 in 2011) and alfalfa (July 18 - September 20 in 2010 and June 5 - August 29 in 2011). Temperatures ranged between 16°C and 35°C and wind speeds were below 15 km/h. Each site was surveyed once by capturing bees along a 50 m long and 1 m wide transect, within the main flowering crops, oilseed rape and sunflower and flowering alfalfa. All species (other than honey bees) were identified in the lab by specialists.

Study no[reference] Crop; Location; year; yield (tonnes.ha^{-1}); price (\$.tonne^{-1}); no. sites; no. wild bees; ratio wild/honey bee

33[12] Oil Seed Rape (*Brassica napus*); France, Brittany; 2007; 2.90; 432.3; 20; 83; 0.76

34[12] Oil Seed Rape (*Brassica napus*); France, Centre; 2007; 2.90; 432.3; 10; 107; 0.83

35[12] Oil Seed Rape (*Brassica napus*); France, Brittany; 2008; 3.32; 495.4; 20; 318; 0.48

36[12] Oil Seed Rape (*Brassica napus*); France, Centre; 2008; 3.32; 495.4; 32; 116; 0.85

Methods: In 2007 and 2008, bees were collected with sweepnets on oilseed rape flowers in two regions of France. Bees were always captured in the first meter of the fields, except in 2008 in Pleine-Fougères where bees were also captured in the middle of the fields. Each field was surveyed three times during the oilseed rape flowering period, with between 3 and 5 days separating surveys. Surveying was conducted under sunny conditions or with scattered clouds. Temperatures ranged between 15 °C and 20 °C with at most a moderate breeze. Depending on the field, a survey round comprised 3 to 12 points. On each point, 3 strikes of a sweepnet were used to catch bees on oilseed rape flowers. All bees were collected and identified in the lab.

Study no[reference] Crop; Location; year; yield (tonnes.ha^{-1}); price (\$.tonne^{-1}); no. sites; no. wild bees; ratio wild/honey bee

37[13] Sunflower (*Helianthus annuus*); Israel; 2009; 5.24; 1399.5; 10; 99; 0.05
38[13] Sunflower (*Helianthus annuus*); Israel; 2010; 5.32; 1603.5; 17; 222; 0.09
39[14] Watermelon (*Citrullus lanatus*); Israel; 2009; 11.12; 430.3; 15; 121; 0.12
40[14] Watermelon (*Citrullus lanatus*); Israel; 2010; 11.93; 385.6; 13; 301; 0.18

Methods: Bees were surveyed on sunflower and watermelon fields in the Judean Foothills in central Israel during crop bloom in May-June, 2009 and 2010. In 2009, 10 sunflower and 17 watermelon fields were surveyed and in 2010, 15 sunflower and 13 watermelon fields were surveyed. Study plots (25 × 25 m) were located at field edges; in some fields (sunflower-9, watermelon-12) an additional interior plot was located 100 m from the edge. Sampling sites were separated by at least 1 km from one another. Field work was conducted under standardized weather conditions (sunny to light overcast skies, temperatures >18 ºC and mean wind velocity <5 m.s^{-1}). Each plot was sampled between one and three times (mostly twice), each time on a separate day. In each sampling day, two sampling sessions (2-3 hours apart) were carried out. Each session included 10 min (or 15 min in sunflower in 2010) of bee netting (the stopwatches were stopped when handling bees that were caught). Bee sampling was conducted between 8:00 and 16:00 in the sunflower study, and between 7:00 and 11:00 in the watermelon study.

Study no[reference] Crop; Location; year; yield (tonnes.ha^{-1}); price (\$.tonne^{-1}); no. sites; no. wild bees; ratio wild/honey bee

41[B.Vaissière] Sunflower (*Helianthus annuus*); France, Rhone-Alpes; 2009; 2.37; 365.4; 5; 169; n.a.

Methods: Non-*Apis* bees were collected in five fields of sunflower for hybrid seed production on both male-fertile (MF) and male-sterile (MS) parental lines (each field had a different pair of parental lines). All fields were located within 20 km east of the town of Montélimar at an altitude ranging from 169 to 270 m. In each field, we established a study site 100 m long over 8 adjacent patterns of MF and MS rows and centered halfway between the center and the edge of the field. Bees were collected with a net over a 30 min interval (not counting handling time), split into 15 min over 100 m of row of MF plants and 15 min over 100 m of rows of MS plants. Bees were surveyed 4 to 8 times over the flowering period, with collections taking place in the morning and in the afternoon on alternate days. All collections took place between 13 and 30 July, 2009 under good weather conditions (vegetation dry, temperature at least 15°C and low to non-existent wind) and between 09:30 and 17:15 local time.

Study no[reference] Crop; Location; year; yield (tonnes.ha^{-1}); price (\$.tonne^{-1}); no. sites; no. wild bees; ratio wild/honey bee

42[M.Park] Apple (*Malus domestica*); USA, New York; 2009; 31.28; 509; 12; 1733; 0.76
43[M.Park] Apple (*Malus domestica*); USA, New York; 2010; 30.46; 556; 9; 724; 0.62
44[M.Park] Apple (*Malus domestica*); USA, New York; 2011; 31.95; 644; 22; 2220; 0.65

Methods: In late April and May from 2009 to 2011, bees visiting apple blossoms were surveyed. Twelve orchards were surveyed in 2009, nine in 2010 and in twenty-two in 2011. Each orchard was surveyed once or twice during the apple bloom, on days with temperature > 15°C between 10:00 and 15:30. We required that there be enough sun to cast a shadow. At each site, multiple transects of 15-minute aerial netting surveys were conducted along blooming tree rows. During each survey, collectors walked a steady pace along 50 m of each side of two-adjacent tree rows and netted all bees observed to be visiting apple blossoms. Transects were spaced, at least, 50 m apart and were placed where trees were in highest bloom, within 150 m from the orchard edge. Distance between orchards was at least 1.9km. The number of timed net collections per site varied according to farm size.

Study no[reference] Crop; Location; year; yield (tonnes.ha^{-1}); price (\$.tonne^{-1}); no. sites; no. wild bees; ratio wild/honey bee

45[R.Winfree] Apple (*Malus domestica*); USA, New Jersey; 2004; 30.36; 300; 16; 151; n.a.

Methods: In April 2004, bees were surveyed in 16 sites in 6-8 commercial apple orchards. At each site, the data collector walked through the orchard, collecting all non-Apis bees visiting apple flowers with a net. One data collection day was conducted per orchard.

Study no[reference] Crop; Location; year; yield (tonnes.ha^{-1}); price (\$.tonne^{-1}); no. sites; no. wild bees; ratio wild/honey bee

46[15] Tomato (*Solanum lycopersicum*); USA, New Jersey/Pennsylvania; 2004; 80.57; 825; 15; 119; 0.99
47[15] Tomato (*Solanum lycopersicum*); USA, New Jersey/Pennsylvania; 2005; 72.55; 917; 13; 86; 1.00

Methods: In June and July, bees were surveyed in tomato fields on 15 study farms in 2004 and 13 study farms in 2005. Surveys used one 50 meter transect per farm within which all data were collected. All non-Apis bees visiting crop flowers were collected by hand net along the entire length of the transect. Total minutes of sampling effort varied across years but was always standardized across all farms within a given year. One sample day per farm was conducted per year. Honey bees were observed visiting flowers in timed samples, but not netted, so data used for honey bees include only observed visitors. Data collection was only conducted on days suitable for bee activity (sunny, partly cloudy or bright overcast; wind speeds <2.5 m.s^{-1}; >18 °C). Bees were identified by professional taxomists.

Study no[reference] Crop; Location; year; yield (tonnes.ha^{-1}); price ($.tonne^{-1}); no. sites; no. wild bees; ratio wild/honey bee
48[16] Cranberry (*Vaccinium macrocarpon*); USA, New Jersey; 2009; 20.13; 937; 16; 1226; 0.2
49[16] Cranberry (*Vaccinium macrocarpon*); USA, New Jersey; 2010; 19.82; 948; 16; 1753; n.a.

Methods: Bees were surveyed in 16 commercial cranberry bogs. Within each bog, two 60 m transects were located; one in the interior of the cranberry bog, one parallel to the edge next to forest. Two sample days per farm were conducted per year, and within each day data sampling was conducted once in the morning and once in the afternoon. All wild bees visiting cranberry flowers within the transect were collected for a total of 60 minutes per collection day. Data were collected from June to July, in each of 2009 and 2010. Honey bees were observed visiting flowers in timed samples, but not collected, so data used for honey bees include only observed visitors. Sampling was only conducted during weather suitable for bee activity (>15 °C, wind <3.5 meters.s^{-1}, not dark overcast). Bees were identified by professional taxomists.

Study no[reference] Crop; Location; year; yield (tonnes.ha^{-1}); price ($.tonne^{-1}); no. sites; no. wild bees; ratio wild/honey bee
50[15] Musk melon (*Cucumis melo*); USA, New Jersey/Pennsylvania; 2004; 27.90; 324; 13; 116; 0.29

Methods: In July, 2004, bees were surveyed for one day each in musk melon fields on 14 study farms. One 50 meter transect was used per farm within which all data were collected. All non-Apis bees visiting crop flowers were collected by hand net along the entire length of the transect for 20 minutes. Sampling was only conducted on days suitable for bee activity (sunny, partly cloudy or bright overcast; wind speeds <2.5 m/s; >18 °C). Honey bees were observed visiting flowers in timed samples, but not collected, so honey bee data includes only observed visitors. Bees were identified by professional taxomists.

Study no[reference] Crop; Location; year; yield (tonnes.ha^{-1}); price ($.tonne^{-1}); no. sites; no. wild bees; ratio wild/honey bee
51[17] Highbush Blueberry (*Vaccinium corymbosum*); USA, New Jersey; 2010; 6.71; 3175; 16; 233; 0.09
52[17] Highbush Blueberry (*Vaccinium corymbosum*); USA, New Jersey; 2011; 6.85; 4057; 16; 396; 0.10

Methods: Bees were surveyed in 16 sites in commercial blueberry fields using one 200 m transect per site. On each site-day, a transect was sampled three times, with 3 site-days per year organized into 3 collection rounds temporally stratefied to span the period of bloom. Sampling was conducted using identical methods in April-May in each of 2 years (2010 and 2011). Each data collection event included 20 minutes of observation and 20 minutes of netting, for a total of 1 hour each of observation and netting per site-day. Honey bees were recorded during timed observation samples, but not netted, so data for honey bees includes only observations. Data were only collected during weather suitable for bee activity, the exact conditions for which shifted over the course of the season, as blueberry is an early spring crop in our region. Bees were identified by professional taxomists.

Study no[reference] Crop; Location; year; yield (tonnes.ha^{-1}); price ($.tonne^{-1}); no. sites; no. wild bees; ratio wild/honey bee
53[18] Watermelon (*Citrullus lanatus*); USA, New Jersey/Pennsylvania; 2004; 29.18; 187; 11; 112; 0.51
54[18] Watermelon (*Citrullus lanatus*); USA, New Jersey/Pennsylvania; 2005; 31.17; 256; 23; 1217; 0.62
55[18] Watermelon (*Citrullus lanatus*); USA, New Jersey/Pennsylvania; 2007; 32.45; 249; 16; 232; 0.54

56[18] Watermelon (*Citrullus lanatus*); USA, New Jersey/Pennsylvania; 2008; 35.71; 276; 18; 792; 0.63
57[18] Watermelon (*Citrullus lanatus*); USA, New Jersey/Pennsylvania; 2010; 34.83; 265; 18; 2048; 0.70

Methods: Bees were surveyed in watermelon fields in the years 2004-2005 and 2007-2009. One 50 meter transect per farm was used within which all data were collected. All non-Apis bees visiting crop flowers were collected by net along the entire length of the transect. Total minutes of sampling effort varied across years (2004, 2005, 2007, 2008, 2010) but was always standardized across all farms within a given year. Each day the transect was sampled at three temporally stratefied samples between 8:00 and 13:00. One sample day per farm per year was conducted in 2007 and 2008, 2 sample days per farm per year were conducted in 2005 and 3 sample days per farm were conducted in 2010. In years with multiple sampling days per year, sampling was organized into rounds, with the rounds temporally stratefied throughout the period of bloom. Data were collected between June and August in each year. Data collection was only conducted on days suitable for bee activity (sunny, partly cloudy or bright overcast; wind speeds <2.5 m/s; >18 C), with a few exceptions. Honey bees were observed visiting flowers in timed samples, but not netted, so data used for honey bees include only observed visitors. Bees were identified by professional taxomists.

Study no[reference] Crop; Location; year; yield (tonnes.ha^{-1}); price (\$.tonne^{-1}); no. sites; no. wild bees; ratio wild/honey bee

58[19] Cranberry (*Vaccinium macrocarpon*); USA, Massachusetts; 1990; 13.68; n.a.; 8; 350; 0.62
59[19] Cranberry (*Vaccinium macrocarpon*); USA, Massachusetts; 1991; 16.72; 1080; 9; 390; 0.57

Methods: Data were extracted from reference (*70*). Bees were surveyed from mid-June to mid-July on cranberry bogs. Eight bogs were surveyed in 1990 and nine in 1991, three of which were the same as in 1990. In each bog, as many bees as possible were captured with either an insect net or jar as the individual collecting moved through the bog over 15 min in 1990 and 10 min in 1991. Collections were carried out three times during cranberry bloom. All bees, including honey bees, foraging on cranberry bloom were collected. The bees were collected and pinned and identified to species in the laboratory.

Study no[reference] Crop; Location; year; yield (tonnes.ha^{-1}); price (\$.tonne^{-1}); no. sites; no. wild bees; ratio wild/honey bee

60[20] Squash (*Cucurbita pepo*); USA, Virginia; 2008; 22.67; 284; 15; 330; 0.99
61[20] Apple (*Malus domestica*); USA, Virginia; 2009; 31.28; 509; 6; 129; 0.94
62[20] Highbush Blueberry (*Vaccinium corymbosum*); USA, Virginia; 2009; 64.65; 2932; 6; 185; 1.00
63[20] Squash (*Cucurbita pepo*); USA, Virginia; 2009; 21.05; 243; 12; 179; 0.91
64[20] Apple (*Malus domestica*); USA, Virginia; 2010; 30.46; 556; 5; 177; 0.73

Methods: Between 2008 and 2010, bees were surveyed on apple, blueberry, and squash flowers on farms in southwest Virginia. Bees were netted at flowers for 15 minutes when temperatures exceeded 21ºC, cloud cover was less than 35%, and wind was less than 3 Beaufort. Exceptions included overcast days when temperatures were relatively warm and honey bees were clearly active, due to the small sampling window for apples and blueberries. Data were used from 15 and 12 squash fields surveyed in 2008 and 2009, respectively, 6 blueberry fields surveyed in 2009 and 6 and 5 apple orchards surveyed in 2009 and 2010, respectively.

Study no[reference] Crop; Location; year; yield (tonnes.ha^{-1}; price (\$.tonne^{-1}; no.sites; no. wild bees; ratio wild/honey bee

65[H.S.Sardiñas] Sunflower (*Helianthus annuus*); USA, California; 2011; 1.57; 648; 11; 203; 0.08

Methods: Eleven hybrid sunflower fields were surveyed in Yolo County, California between June and August, 2011. Bees were collected on male-fertile and male-sterile flowers between 8:00 and 14:00 on sunny days with temperatures exceeding 18°C and wind speeds below 3 m.s^{-1}. In each field, bees were netted 10, 50, 100 and 200 m from the field edge for 16 minutes at each distance, stopping the clock during specimen handling. Honey bees were counted in visual surveys. Species were identified to the lowest taxonomic level possible.

Study no[reference] Crop; Location; year; yield (tonnes.ha^{-1}); price (\$.tonne^{-1}); no. sites; no. wild bees; ratio wild/honey bee

66[A.M.Klein] Almond (*Prunus dulcis*); USA, California; 2009; 39.89; 3638; 15; 130; 0.22

Methods: In February and March, 2009, bees were surveyed in 15 almond orchards in California. Six experienced entomologists conducted the flower observations under sunny to lightly overcast conditions, when temperatures exceeded 13°C and when wind speeds were below 2.5 $m \cdot s^{-1}$. In each orchard we observed flower visitors on five trees at the orchard edge closest to semi-natural habitat. At each tree, eight groups of flowers were observed for three times 20 seconds each, two each in the inner top, inner bottom, outer top and outer bottom quadrants of the tree (total of around 13 min per orchard). Species were identified mainly by close observations of the flower bundles or caught for identification in the lab.

Study no[reference] Crop; Location; year; yield (tonnes.ha^{-1}); price (\$.tonne^{-1}); no. sites; no. wild bees; ratio wild/honey bee

67[21, L.Button] H. blueberry (*V. corymbosum*); Canada, British Columbia; 2011; 2.73; 1825.6; 26; 657; 0.25

68[21, L.Button] H. blueberry (*V. corymbosum*); Canada, British Columbia; 2012; 3.28; n.a.; 37; 492; 0.17

Methods: In 2011 and 2012, bees were observed on four highbush blueberry varieties. Fields were sampled 2-5 times in each year at varying times of day (morning, mid day, and afternoon) during peak blueberry bloom. Observations for Duke and Bluecrop varieties were conducted along three transects per field, with ten 1 minute observation periods per transect. Draper and Liberty sites only contained two transects. Observations were conducted on either sunny days with temperatures >14^{0}C, or cloudy days with temperatures > 17^{0}C.

Study no[reference] Crop; Location; year; yield (tonnes.ha^{-1}); price (\$.tonne^{-1}); no. sites; no. wild bees; ratio wild/honey bee

69[22] Tomato (*Solanum lycopersicum*); USA, California; 2001; 66.75; 661; 11; 708; 0.99

Methods: In July and August, 2001, bees were surveyed in 11 tomato fields in northern California. In each tomato field, bees were surveyed by walking transects at the rate of 10 m/min, covering each row twice, once in each direction, and recording all bee visits to tomato flowers. In small fields, transects were walked along all rows. In larger fields, surveys were carried out at up to four transects, each 80m long. Each field was sampled between 8:30 and 12:30 on three different days, in the early, mid, and late morning, respectively.

Study no[reference] Crop; Location; year; yield (tonnes.ha^{-1}); price (\$.tonne^{-1}); no. sites; no. wild bees; ratio wild/honey bee

70[A.R.Sciligo] Strawberry (*Fragaria x ananassa*); USA, California; 2011; 56.45; n.a.; 7; 179; 0.61

71 [A.R.Sciligo] Strawberry (*Fragaria x ananassa*); USA, California; 2012; 58.96; n.a.; 17; 458; 0.44

Methods: In 2011 and 2012, bees were surveyed in organic strawberry crops in northern California. In 2011, surveys were conducted once each at 7 sites. In 2012, surveys were conducted twice, once in spring and once in summer at each of 17 sites (though one site was dropped from summer sampling because strawberries were pulled up). Bees were collected via netting in good weather conditions (at least partially sunny with low wind between 0.4 and 3.5 $m.s^{-1}$, and temperatures above 14°C). At each site, six 10-minute sample periods took place in the same good weather conditions. Clocks were stopped when handling specimens. All bees collected were identified by a professional taxonomist to the lowest taxonomic level possible. For a number of *Lasioglossum* species, only females could be identified to species level. We therefore allocated all unidentified *Lasioglossum* males in proportion to the numbers that were identified as female *Lasioglossum* specimens. Honeybees were not collected and observed counts were recorded.

Study no[reference] Crop; Location; year; yield (tonnes.ha^{-1}); price (\$.tonne^{-1}); no. sites; no. wild bees; ratio wild/honey bee

72[N.Williams] Watermelon (*Citrullus lanatus*); USA, California; 2010; 34.83; 265; 21; 551; n.a.

73[N.Williams] Watermelon (*Citrullus lanatus*); USA, California; 2011; 34.36; 306; 25; 477; n.a.

Methods: In 2010 and 2011 bees were netted during standardized time periods on production watermelon fields in central California. Some farms were sampled in both years but never the same field. Within a year each site was visited three times during peak bloom at 4-5 day intervals. Sites were visited between 8 June and 3 August, 2010 and between 22 June and 16 August, 2011 during sunny conditions with temperatures between 22 °C and 33 °C and with wind speeds below 3 $m.s^{-1}$. On each sampling date, bees were netted during four 10-minute periods during the day along the same 50 m transect (40 min total). All specimens were collected and identified to species in the lab. For a

number of *Lasioglossum* species, only females could be identified to species level. We therefore allocated all unidentified *Lasioglossum* males in proportion to the numbers that were identified as female *Lasioglossum* specimens.

Study no[reference] Crop; Location; year; yield (tonnes.ha^{-1}); price (\$.tonne^{-1}); no. sites; no. wild bees; ratio wild/honey bee
74[23] Coffee (*Coffea arabica*); Indonesia, Sulawesi; 2001; 0.43; 762.8; 24; 1769; 0.87
75[24] Coffee (*Coffea canephora*); Indonesia, Sulawesi; 2001; 0.43; 762.8; 15; 2113; 0.93

Methods: In 2000 to January, 2001 bees visitating highland coffee flowers were surveyed in 24 agroforestry systems and bees visiting lowland coffee flowers were surveyed in 15 agroforestry systems. Agroforestry systems were dominated by coffee and cacao and located in the buffer zone of the Lore-Lindu National Park in Central Sulawesi. Bee flower visitation was observed for 25 minutes on a full-blooming coffee plant per agroforestry system and this was repeated three times for a total of 75 minutes of observation time for each of the 24 agroforestry systems. Each day, a different full-blooming coffee plant than that used the day before was observed. Sampling was carried out between 9:00 and 14:00 on sunny to slightly overcast days. Easily recognizable species were identified in the field while others were collected and identified with the help of trained locals in the lab.

Study no[reference] Crop; Location; year; yield (tonnes.ha^{-1}); price (\$.tonne^{-1}); no. sites; no. wild bees; ratio wild/honey bee
76[25] Onion (*Allium cepa*); New Zealand, South Island; 2004; n.a.; n.a.; 11; 1085; 0.14
77[25] Onion (*Allium cepa*); New Zealand, South Island; 2005; n.a.; n.a.; 11; 897; 0.10
78[B.Howlett] Onion (*Allium cepa*); New Zealand, South Island; 2006; n.a.; n.a.; 9; 216; 0.07
79[B.Howlett] Onion (*Allium cepa*); New Zealand, South Island; 2007; n.a.; n.a.; 8; 161; 0.07
80[B.Howlett] Onion (*Allium cepa*); New Zealand, South Island; 2008; n.a.; n.a.; 4; 163; 0.12

Methods: From 2004 to 2008 bees were surveyed on 4-11 onion fields. Each field contained 5 sample points (4 at each corner and one in the centre). At each observation point, bee counts were conducted on 75 male sterile and 75 male fertile flowering umbels, each containing more than 30 open flowers. Bee counts were carried out by slowly walking along each row of flowering umbels and recording individuals on a spreadsheet at the lowest taxonomic level possible. Each observation point was surveyed three times during the day (10-11 am, 12-1 pm and 2-3 pm). Hand collection of bees using vials, containers and nets were also utilised to help identify bees to species level.

Study no[reference] Crop; Location; year; yield (tonnes.ha^{-1}); price (\$.tonne^{-1}); no. sites; no. wild bees; ratio wild/honey bee
81[B.Howlett] Carrot (*Daucus carota*); New Zealand, South Island; 2010; n.a.; n.a.; 4; 149; 0.23

Methods: In 2010 bees were surveyed on 4 carrot fields. In each carrot field three observation points (two corner and one centre point) were marked. At each observation point, observations were conducted on 150 carrot inflorescences within a 5 metre radius. Three observations were carried out during the day at each point. These were at 10-11 am, 12-1 pm and 2-3 pm. Only umbels with more than 30% of flowers open were observed. Umbels were examined along rows within the confines of the marked observation points. Approximately 15 minutes were required to complete the observations at each point and 60 minutes to complete observations across an entire field. When the identity of the bee species was unknown, specimens were collected using vials, containers and nets for identification in the laboratory.

Study no[reference] Crop; Location; year; yield (tonnes.ha^{-1}); price (\$.tonne^{-1}); no. sites; no. wild bees; ratio wild/honey bee
82[26] Sunflower (*Helianthus annuus*); South Africa, Limpopo; 2009; 1.26; 341.8; 33; 802†; n.a.

Methods: In March and April, 2009 bees were surveyed in commercial sunflower farms by capturing all visitors of flowerheads. Surveys were conducted in 33 plots (4 x 4 m, sunflower density of 10 plants m2) within sunflower fields and plots were at least 350 m away from each other. Each plot was surveyed on two different days (once in the morning and once in the afternoon) during the week of peak flowering. In each survey, three locations (one for each of three observers) were randomly selected within the plot and all the sunflower heads that could be reached were observed for 4 minutes, during which all insects that touched the reproductive parts of the flowers were recorded. In

total, each plot was observed for 24 minutes. All bees collected were identified to the lowest possible taxonomic level by an expert entomologist.

Study no[reference] Crop; Location; year; yield (tonnes.ha^{-1}); price (\$.tonne^{-1}); no. sites; no. wild bees; ratio wild/honey bee

83[J.F.Colville] Sunflower (*Helianthus annuus*); South Africa, Limpopo; 2010; 1.23; 404.0; 10; 3652†; n.a.

Methods: During 16-23 March, 2011 insect pollinators were surveyed on ten commercial sunflower farms. Five field study sites were selected adjacent to natural vegetation (<200 m) and five were selected at a distance >2000 m from natural vegetation. On each field study site, 100 flower heads in each of four parallel transects spaced 20 m apart (total 400 flower heads) were surveyed in the morning (09:00 – 12:00) and afternoon (14:00 – 16:00). Surveys were conducted by walking along a transect between rows of plants and recording the number of insect pollinators seen on individual flower heads, one by one. Voucher specimens for all insect flower visitors that touched the reproductive structures of surveyed sunflower heads were collected. From this, all bee specimens were identified to the lowest possible taxonomic level.

Study no[reference] Crop; Location; year; yield (tonnes.ha^{-1}); price (\$.tonne^{-1}); no. sites; no. wild bees; ratio wild/honey bee

84[R.Veldtman] Apple (*Malus domestica*); South Africa, Western Cape; 2011; 34.32; 515.2; 10; 3133†; n.a.

Methods: From 7-13 October, 2011 bees were surveyed on Royal Gala Apples in the Grabouw and Viliersdorp areas of the Western Cape. Five fields were within 200 m of natural vegetation while five others were at least 2 km away from natural vegetation. All sites stocked managed honeybees at an average of two hives per hectare. Each crop was surveyed in the morning and afternoon for one good weather day. On each survey, one side of eight trees along a transect (trees spaced 5 m apart) were scanned for five minutes each and bee species were recorded and voucher specimens collected (800 minutes of total observation time; average of 758.5 +/- 265.1 [1SD] open flowers per apple tree). All pollinators visiting flowers were identified to the lowest possible taxonomic level by an expert entomologist.

Study no[reference] Crop; Location; year; yield (tonnes.ha^{-1}); price (\$.tonne^{-1}); no. sites; no. wild bees; ratio wild/honey bee

85[M.Brand] Onion (*Allium cepa*); South Africa; 2009; n.a.; n.a.; 4; 1815†; n.a.

86 [M.Brand] Onion (*Allium cepa*); South Africa; 2010; n.a.; n.a.; 8; 659†; n.a.

Methods: In 2009 and 2010 bees were surveyed on hybrid onion seed crops in South Africa. Different crop fields were used each year. Each crop was surveyed for one good weather day during the blooming season from 23 October to 11 November, 2009 and from 18 October to 30 November, 2010). Field workers made observations during four data collection periods spread over 2 hour intervals between 9:00 and 16:00 on each observation day. Observations were replicated five times on both male-fertile and male-sterile rows during each collection period. Four neighbouring umbels in at least 50% bloom were selected and observed for 4 minutes. Bees visiting the umbels were collected and identied in the lab.

Study no[reference] Crop; Location; year; yield (tonnes.ha^{-1}); price (\$.tonne^{-1}); no. sites; no. wild bees; ratio wild/honey bee

87[27] Coffee (*Coffea arabica*); Costa Rica, Perez Zeledon; 2001; 1.33; 521.5; 12; 279; 0.49

88[27] Coffee (*Coffea arabica*); Costa Rica, Perez Zeledon; 2002; 1.24; 605.1; 16; 339; 0.72

Methods: In 2001 and 2002, bees were surveyed on highland coffee bushes (*Coffea arabica*, var. Caturra). In 2001, 12 sites were surveyed, and in 2002 16 sites were surveyed (8 of which had been surveyed in 2001). At each site, on each day in which coffee was in flower, 2 simultaneous samples of flower visitors were taken. Each sample involved recording each visitor and the number of flowers visited for 10 minutes on an area of one bush comprising approximately 250 flowers.

Study no[reference] Crop; Location; year; yield (tonnes.ha^{-1}); price (\$.tonne^{-1}); no. sites; no. wild bees; ratio wild/honey bee

89[B.F.Viana] Passion fruit (*Passiflora edulis*); Brazil, Bahia; 2005; n.a.; n.a.; 16; 1049; 0.75

Methods: In 2005, bees were surveyed in 16 sites with passion fruit crops in São Francisco Valley region. In each field, bees were surveyed in a 50 m long transect, laid within the crop field, with a mean of 90 flowers observed for 15 minutes during three times on three different days. Each

crop was surveyed by experienced biologists. All flower visiting wild bees were collected for identification by specialists.

Study no[reference] Crop; Location; year; yield (tonnes.ha^{-1}); price (\$.tonne^{-1}); no. sites; no. wild bees; ratio wild/honey bee

90[28] Coffee (*Coffea arabica/robusta*); Mexico, Ciapas; 2006; 0.37; 244.9; 13; 140; 0.32

Methods: In April 2006, bees were surveyed on coffee flowers at 13 different sites in cofffee plantations in Nueva Alemania in the southern highlands of Chiapas. In the study region, Coffea arabica and Coffea robusta are planted (approximately 4000 coffee bushes per hectare) under a canopy of overstorey trees. Surveys were conducted in 15 minute periods between 8:00 and 14:00. At each site, four fully flowering branches (minimum of 20 blossoms) were randomly chosen from a randomly selected cofffe bush. During observation periods, the identity of the visitor was noted and, when possible, bees were captured after the observation period for identification.

† Includes managed bees hired for pollination as well.

Supplementary Table 2 | The top 100 bee species with the highest mean contribution to crop production value and their occurrence in different studies and crops. Mean contribution to crop production is based on the 53 studies for which contribution to production value could be calculated (i.e. data on crop production value and the relative contribution between wild and managed bees were available). Occurrences also include crops and studies for which no contribution to crop production value could be calculated.

Rank	Species	Biogeographic region of study	Mean contributed value in region ($.ha^{-1})	95% CI interval	Maximum contributed value in region ($.ha^{-1})
1	*Bombus impatiens*	Eastern North-America	963.0	645-1279	2800
2	*Bombus terrestris/lucorum* †	Europe	425.0	122-892	4532
3	*Bombus lapidarius*	Europe	366.0	59-896	5707
4	*Anthophora urbana*	Western North-America	314.0	0-930	1240
5	*Andrena chrysosceles*	Europe	299.0	14-736	4256
6	*Andrena vicina*	Eastern North-America	296.0	116-511	1865
7	*Andrena flavipes*	Europe	289.0	60-548	2185
8	*Augochlora pura*	Eastern North-America	268.8	164-402	974
9	*Andrena haemorrhoa*	Europe	268.0	43-583	2969
10	*Andrena crataegi*	Eastern North-America	254.0	47-542	1823
11	*Bombus vosnesenskii*	Western North-America	244.0	20-674	886
12	*Andrena carantonica*	Europe	218.0	17-541	3326
13	*Andrena carlini*	Eastern North-America	210.0	35-471	2264
14	*Lasioglossum versatum*	Eastern North-America	206.8	107-320	722
15	*Bombus bimaculatus*	Eastern North-America	180.0	68-313	1065
16	*Andrena cerasifolii*	Western North-America	177.0	0-556	741
17	*Peponapis pruinosa*	Eastern North-America	160.8	31-359	1547
18	*Ceratina calcarata/dupla/mikmaqi* †	Eastern North-America	155.9	71-250	666
19	*Lasioglossum imitatum*	Eastern North-America	138.2	40-270	1010
20	*Andrena barbara*	Eastern North-America	136.6	0-344	1435
21	*Osmia cornifrons*	Eastern North-America	133.5	18-264	906
22	*Andrena regularis*	Eastern North-America	132.0	0-286	1023
23	*Bombus griseocollis*	Eastern North-America	118.9	55-205	679
24	*Xylocopa virginica*	Eastern North-America	113.3	49-195	599
25	*Lasioglossum hitchensi* [1]	Eastern North-America	107.8	62-160	314
26	*Lasioglossum leucocomum/pilosum* †	Eastern North-America	95.7	40-161	469
27	*Bombus melanopygus*	Western North-America	89.1	0-216	252
28	*Lasioglossum (Evyl.)* sp. E (Calif.)	Western North-America	88.0	0-260	347
29	*Bombus flavifrons*	Western North-America	83.1	0-246	329
30	*Bombus pascuorum*	Europe	81.5	19-155	665
31	*Augochlorella aurata*	Eastern North-America	80.4	38-133	389
32	*Osmia lignaria*	Eastern North-America	75.8	2-188	1005
33	*Andrena nasonii*	Eastern North-America	75.4	8-187	949
34	*Panurginus gracilis*	Western North-America	73.5	0-225	300
35	*Halictus confusus*	Eastern North-America	72.7	27-140	606
36	*Andrena miserabilis*	Eastern North-America	71.2	17-140	500
37	*Colletes inaequalis*	Eastern North-America	67.8	20-137	537
38	*Bombus mixtus*	Western North-America	64.2	0-189	252
39	*Bombus perplexus*	Eastern North-America	57.3	23-104	395
40	*Bombus sandersoni*	Eastern North-America	49.9	4-109	466
41	*Bombus vagans*	Eastern North-America	46.3	2-130	756
42	*Osmia taurus*	Eastern North-America	45.3	0-113	599
43	*Andrena hippotes*	Eastern North-America	44.9	13-98	341
44	*Melissodes bimaculata*	Eastern North-America	41.1	16-69	172
45	*Andrena fulva*	Europe	40.8	8-85	396
46	*Andrena dorsata*	Europe	40.8	5-89	475
47	*Lasioglossum nymphaearum*	Eastern North-America	38.3	8-78	306
48	*Andrena rugosa*	Eastern North-America	34.6	9-67	223
49	*Andrena pruni*	Eastern North-America	34.6	3-74	302
50	*Andrena perplexa*	Eastern North-America	34.0	11-65	227

†Consisting of two or more indistinguishable species; [1]species originally identified as *L. mitchelli*

Supplementary Table 2 | Continued.

Rank	Species	Biogeographic region of study	Mean contributed value in region	95%CI interval	Maximum contributed value in region
51	*Andrena w-scripta*	Eastern North-America	33.5	0-90	550
52	*Lasioglossum weemsi*	Eastern North-America	33.2	12-62	194
53	*Lasioglossum tegulare*	Eastern North-America	31.5	11-59	239
54	*Halictus rubicundus*	Eastern North-America	30.8	9-66	320
55	*Andrena forbesii*	Eastern North-America	30.0	7-57	209
56	*Lasioglossum calceatum*	Europe	29.2	4-74	475
57	*Agapostemon virescens*	Eastern North-America	28.7	0-72	332
58	*Andrena bradleyi*	Eastern North-America	28.6	0-73	305
59	*Bombus terricola*	Eastern North-America	26.9	0-81	533
60	*Bombus affinis*	Eastern North-America	24.3	0-72	481
61	*Lasioglossum malachurum*	Europe	23.8	6-45	169
62	*Bombus hypnorum*	Europe	21.0	4-42	197
63	*Colletes validus*	Eastern North-America	20.4	0-57	323
64	*Melitta americana*	Eastern North-America	20.0	0-56	291
65	*Osmia bicornis*	Europe	19.8	1-45	259
66	*Bombus pratorum*	Europe	19.1	6-35	111
67	*Andrena imitatrix*	Eastern North-America	18.7	3-40	177
68	*Andrena dunningi*	Eastern North-America	18.2	3-36	151
69	*Lasioglossum paradmirandum*	Eastern North-America	18.0	7-30	94
70	*Osmia pumila*	Eastern North-America	17.8	2-39	151
71	*Augochloropsis metallica*	Eastern North-America	17.8	3-41	193
72	*Andrena illini*	Eastern North-America	17.6	0-39	151
73	*Triepeolus remigatus*	Eastern North-America	17.5	2-38	145
74	*Lasioglossum illinoense*	Eastern North-America	17.1	1-45	271
75	*Andrena nitida*	Europe	17.1	4-35	190
76	*Nomada luteoloides*	Eastern North-America	16.8	1-39	200
77	*Andrena fenningeri*	Eastern North-America	16.5	0-44	227
78	*Andrena minutula*	Europe	16.1	2-35	190
79	*Eucera lunata*	Western North-America	15.9	0-47	63
80	*Lasioglossum zephyrum*	Eastern North-America	15.6	3-31	123
81	*Andrena mariae*	Eastern North-America	15.2	0-45	302
82	*Halictus ligatus*	Eastern North-America	14.5	5-26	67
83	*Bombus hortorum*	Europe	13.7	3-28	137
84	*Lasioglossum callidum*	Eastern North-America	13.5	4-25	74
85	*Ceratina strenua*	Eastern North-America	13.4	3-27	84
86	*Nomada maculata*	Eastern North-America	13.4	0-34	200
87	*Andrena mandibularis*	Eastern North-America	13.3	0-31	141
88	*Lasioglossum politum*	Europe	13.0	3-27	98
89	*Andrena tridens*	Eastern North-America	12.9	0-31	183
90	*Lasioglossum (Evyl.)* sp. F (Calif.)	Western North-America	12.3	0-36	47
91	*Lasioglossum morio*	Europe	12.3	0-32	190
92	*Lasioglossum foxii*	Eastern North-America	11.9	2-25	76
93	*Colletes thoracicus*	Eastern North-America	11.7	1-26	133
94	*Andrena milwaukeensis*	Eastern North-America	11.6	0-25	94
95	*Osmia virga*	Eastern North-America	11.5	0-32	200
96	*Lasioglossum leucozonium*	Eastern North-America	11.2	1-25	111
97	*Andrena cressonii*	Eastern North-America	11.0	1-24	95
98	*Bombus fervidus*	Eastern North-America	11.0	3-21	67
99	*Andrena cineraria*	Europe	9.5	4-16	48
100	*Andrena commoda*	Eastern North-America	9.1	0-22	110

Supplementary Table 2 | Continued.

Rank	Species	Alfalfa	Almond	Apple	Carrot	Coffee	Cranberry	Field Bean	H. Blueberry	Leek	Musk melon	Oil Seed Rape	Onion	Passion fruit	Pear	Red Clover	Squash	Strawberry	Sunflower	Tomato	Watermelon	Total no. crops	Total no. studies
1	*Bombus impatiens*			6			4		3		1						2			2	5	**7**	**23**
2	*Bombus terrestris/lucorum* †	9		2	1			2		1		10	5		2	5		2	5			**11**	**44**
3	*Bombus lapidarius*	9		2				2		1		10			2	5		2	4			**9**	**37**
4	*Anthophora urbana*																		1	1	1	**3**	**3**
5	*Andrena chrysosceles*			2								5			2			2				**4**	**11**
6	*Andrena vicina*			6			4		3													**3**	**13**
7	*Andrena flavipes*	9		2						1		10			2				5			**6**	**29**
8	*Augochlora pura*			6			2		3		1						2			2	5	**7**	**21**
9	*Andrena haemorrhoa*			2				2				9			2			1				**5**	**16**
10	*Andrena crataegi*			4			1		1													**3**	**6**
11	*Bombus vosnesenskii*		1						2									2	1	1	2	**6**	**9**
12	*Andrena carantonica*			2				2				3			2			1				**5**	**10**
13	*Andrena carlini*			6			1		2													**3**	**9**
14	*Lasioglossum versatum*			4			2		2		1						2			2	5	**7**	**18**
15	*Bombus bimaculatus*			6			4		3		1						2				4	**6**	**20**
16	*Andrena cerasifolii*		1																			**1**	**1**
17	*Peponapis pruinosa*										1						2				6	**3**	**9**
18	*Ceratina calcarata/dupla/mikmaqi* †			7			3		2		1										5	**5**	**18**
19	*Lasioglossum imitatum*			2													2			2	5	**4**	**11**
20	*Andrena barbara*			3					1													**2**	**4**
21	*Osmia cornifrons*			6					1													**2**	**7**
22	*Andrena regularis*			3																		**1**	**3**
23	*Bombus griseocollis*			5			4		3								2			1	3	**6**	**18**
24	*Xylocopa virginica*			5			3		3		1										3	**5**	**15**
25	*Lasioglossum hitchensi* [1]			4					1								2			2	5	**5**	**14**
26	*Lasioglossum leucocomum/pilosum* †			1			2		3								2			1	4	**6**	**13**
27	*Bombus melanopygus*		1						2									1				**3**	**4**
28	*Lasioglossum (Evyl.)* sp. E (Calif.)		1															1				**2**	**2**
29	*Bombus flavifrons*								2													**1**	**2**
30	*Bombus pascuorum*	7		2				2		1		6			2	5		2	4			**9**	**31**
31	*Augochlorella aurata*			3			2		3		1						2			2	4	**7**	**17**
32	*Osmia lignaria*			5					1													**2**	**6**
33	*Andrena nasonii*			5																		**1**	**5**
34	*Panurginus gracilis*		1																			**1**	**1**
35	*Halictus confusus*			3			2				1						1			1	5	**6**	**13**
36	*Andrena miserabilis*			6																		**1**	**6**
37	*Colletes inaequalis*			5					3													**2**	**8**
38	*Bombus mixtus*								2													**1**	**2**
39	*Bombus perplexus*			5			4		3		1						1				1	**6**	**15**
40	*Bombus sandersoni*			4			1		1													**3**	**6**
41	*Bombus vagans*			1			4		1								1				1	**5**	**8**
42	*Osmia taurus*			3					1													**2**	**4**
43	*Andrena hippotes*			4																		**1**	**4**
44	*Melissodes bimaculata*										1						2				5	**3**	**8**
45	*Andrena fulva*			2								4			2			1				**4**	**9**
46	*Andrena dorsata*			2								5			2							**3**	**9**
47	*Lasioglossum nymphaearum*			2													1			2	4	**4**	**9**
48	*Andrena rugosa*			5																		**1**	**5**
49	*Andrena pruni*			4					1													**2**	**5**
50	*Andrena perplexa*			6					1													**2**	**7**

†Consisting of two or more indistinguishable species; [1]species originally identified as *L. mitchelli*

Supplementary Table 2 | Continued.

Rank	Species	Alfalfa	Almond	Apple	Carrot	Coffee	Cranberry	Field Bean	H. Blueberry	Leek	Musk melon	Oil Seed Rape	Onion	Passion fruit	Pear	Red Clover	Squash	Strawberry	Sunflower	Tomato	Watermelon	Total no. crops	Total no. studies
51	*Andrena w-scripta*			3																		**1**	**3**
52	*Lasioglossum weemsi*			3					1											2	4	**4**	**10**
53	*Lasioglossum tegulare*																1			2	5	**3**	**8**
54	*Halictus rubicundus*	3		4			3	1			1	1						1			4	**8**	**18**
55	*Andrena forbesii*			6																		**1**	**6**
56	*Lasioglossum calceatum*	6		2								2			2			2	1			**6**	**15**
57	*Agapostemon virescens*																2				2	**2**	**4**
58	*Andrena bradleyi*								2													**1**	**2**
59	*Bombus terricola*			1			2															**2**	**3**
60	*Bombus affinis*						2															**1**	**2**
61	*Lasioglossum malachurum*	9		1						1		2			1				6		2	**7**	**22**
62	*Bombus hypnorum*			2				1				2			1	2		1	1			**7**	**10**
63	*Colletes validus*								2													**1**	**2**
64	*Melitta americana*						4															**1**	**4**
65	*Osmia bicornis*			2								5			1			1				**4**	**9**
66	*Bombus pratorum*			2				2				5			1	3		2				**6**	**15**
67	*Andrena imitatrix*			5			2		1													**3**	**8**
68	*Andrena dunningi*			6																		**1**	**6**
69	*Lasioglossum paradmirandum*			3																1	5	**3**	**9**
70	*Osmia pumila*			5			2		2													**3**	**9**
71	*Augochloropsis metallica*						3		1								1			1	2	**5**	**8**
72	*Andrena illini*			2					1													**2**	**3**
73	*Triepeolus remigatus*										1						1				3	**3**	**5**
74	*Lasioglossum illinoense*																1				3	**2**	**4**
75	*Andrena nitida*			2				1				7			2			1				**5**	**13**
76	*Nomada luteoloides*			4					1													**2**	**5**
77	*Andrena fenningeri*			2					2													**2**	**4**
78	*Andrena minutula*			2				1		1		3			2			1				**6**	**10**
79	*Eucera lunata*		1																			**1**	**1**
80	*Lasioglossum zephyrum*			1					1								2			1	2	**5**	**7**
81	*Andrena mariae*			1																		**1**	**1**
82	*Halictus ligatus*			1					1		1								1	1	5	**6**	**10**
83	*Bombus hortorum*	7		2				2				3			1	5			2			**7**	**22**
84	*Lasioglossum callidum*								1								2			1	2	**4**	**6**
85	*Ceratina strenua*																				5	**1**	**5**
86	*Nomada maculata*			1					1													**2**	**2**
87	*Andrena mandibularis*			4					1													**2**	**5**
88	*Lasioglossum politum*																		4		2	**2**	**6**
89	*Andrena tridens*			2																		**1**	**2**
90	*Lasioglossum (Evyl.)* sp. F (Calif.)		1																			**1**	**1**
91	*Lasioglossum morio*	3										2			2							**3**	**7**
92	*Lasioglossum foxii*			5					1													**2**	**6**
93	*Colletes thoracicus*			1					3													**2**	**4**
94	*Andrena milwaukeensis*			4																		**1**	**4**
95	*Osmia virga*						2		1													**2**	**3**
96	*Lasioglossum leucozonium*	6		3													2		1		1	**5**	**13**
97	*Andrena cressonii*			3			2		1													**3**	**6**
98	*Bombus fervidus*			1					1		1									2	1	**5**	**6**
99	*Andrena cineraria*			2				1				8						1				**4**	**12**
100	*Andrena commoda*			3																		**1**	**3**

Supplementary Table 3 | The species that were identified as dominant bee crop pollinators in the 90 studies. Listed are all species that make up at least five per cent of all individuals of wild bees on crop flowers in at least one study.

Species	Species	Species
**Brazil**	*Nomada lathburiana*	*Colletes inaequalis*
Trigona spinipes	*Osmia bicolor*	*Colletes validus*
Xylocopa frontalis	*Rhophitoides canus*	*Dialictus admirandus*
Xylocopa grisescens	**Indonesia**	*Habropoda laboriosa*
**Costa Rica**	*Apis dorsata binghami*	*Halictus confusus*
Nannotrigona mellaria	*Apis nigrocinta*	*Lasioglossum hitchensi*[1]
Plebeia frontalis	*Heriades* sp. 1	*Lasioglossum illinoense*
Plebeia jatiformis	*Megachile (Creightonella) atrata*[5]	*Lasioglossum imitatum*
Tetragonisca angustula[3]	*Nomia thoracica*[6]	*Lasioglossum leucocomum/pilosum*†
Trigona sp. 1	*Trigona (Heterotrigona)* sp. 2	*Lasioglossum nymphaearum*
Trigona fulviventris	*Trigona (Lepidotrigona) terminata*	*Lasioglossum versatum*
**Europe**	**Mexico**	*Lasioglossum weemsi*
Andrena carantonica	*Dialictus* sp. 2	*Melitta americana*
Andrena chrysosceles	*Halictus hesperus*	*Osmia cornifrons*
Andrena cineraria	*Nannotrigona perilampoides*[4]	*Osmia lignaria*
Andrena decipiens	*Plebeia* sp. 2	*Peponapis pruinosa*
Andrena distinguenda	*Scaptotrigona mexicana*	*Xylocopa virginica*
Andrena dorsata	**New Zealand**	**Western North America**
Andrena flavipes	*Bombus terrestris*	*Andrena cerasifolii*
Andrena haemorrhoa	*Lasioglossum sordidum*	*Anthophora urbana*
Andrena helvola	*Leioproctus fulvescens*	*Bombus flavifrons*
Andrena labialis	*Leioproctus huakiwi*	*Bombus melanopygus*
Andrena lagopus	*Leioproctus waipounamu*	*Bombus mixtus*
Andrena nigroaenea	**South Africa**	*Bombus vosnesenskii*
Andrena nitida	*Apis mellifera*	*Diadasia enavata*
Andrena ovatula	**Eastern North America**	*Halictus tripartitus*
Andrena subopaca	*Agapostemon virescens*	*Lasioglossum (Dialictus)* sp. AS-2
Anthidium septemspinosum	*Andrena barbara*	*Lasioglossum (Dialictus)* sp. D
Bombus hortorum	*Andrena bradleyi*	*Lasioglossum (Evylaeus)* sp. E
Bombus lapidarius	*Andrena carlini*	*Lasioglossum imbrex*[2]
Bombus pascuorum	*Andrena crataegi*	*Lasioglossum incompletum*
Bombus pratorum	*Andrena miserabilis*	*Lasioglossum kincaidii*
Bombus subterraneus	*Andrena morrisonella*	*Melissodes agilis*
Bombus terrestris/lucorum†	*Andrena nasonii*	*Melissodes lupina*
Ceratina cucurbitina	*Andrena nuda*	*Panurginus gracilis*
Ceratina mandibularis	*Andrena perplexa*	*Svastra obliqua*
Eucera clypeata	*Andrena regularis*	
Halictus resurgens	*Andrena vicina*	
Halictus rubicundus	*Andrena w-scripta*	
Halictus scabiosae	*Augochlora pura*	
Halictus simplex	*Augochlorella aurata*	
Halictus tetrazonianellus	*Augochloropsis metallica*	
Hylaeus punctulatissimus	*Bombus affinis*	
Hylaeus taeniolatus	*Bombus bimaculatus*	
Lasioglossum malachurum	*Bombus griseocollis*	
Lasioglossum pauxillum	*Bombus impatiens*	
Lasioglossum politum	*Bombus perplexus*	
Lasioglossum subhirtum	*Bombus terricola*	
Lasioglossum xanthopus	*Bombus vagans vagans*	
Melitta leporina	*Ceratina calcarata/dupla/mikmaqi*†	

† Consisting of two or more indistinguishable species; ‡ Including West Coast observations; [1]Species originally identified as *L. mitchelli*; [2]Species originally identified as *L. tegulariforme*; [3]Originally identified as *Trigona (tetragonisca) angustula*; [4]Originally identified as *Nannotrigona testaceicornis*; [5]Originally identified as *Creightonella frontalis atrata*; [6]Originally identified as *Nomia (Thoraconomia) thoracica.*

Supplementary Table 4 | The relationship between flower visitation frequency and crop pollination. For each crop and year we show the number of pollinator species groups analysed (n; number of species are in parentheses), the Pearson correlation (r) between visitation frequency and total pollination, the correlation between mean per visit pollen deposition and total pollination, the correlation between visitation frequency and mean per visit pollen deposition, and the ratio of the standard deviations of the logarithm of the visitation frequency and logarithm of the per visit pollen deposition (the parameter R of reference 28).

Crop, year	n	r visitation-total pollination	r mean per visit deposition - total pollination	r visitation-mean per visit deposition	SD (log visitation) / SD (log per visit deposition)
H. Blueberry, 2010	8 (23)	0.89	0.07	-0.21	1.34
H. Blueberry, 2011	8 (30)	0.86	-0.06	-0.33	1.59
Cranberry, 2009	11 (48)	0.79	0.51	0.02	1.91
Cranberry, 2010	10 (40)	0.93	0.41	0.11	1.73
Tomato, 2004	9 (19)	0.97	-0.56	-0.72	6.31
Tomato, 2005	4 (17)	0.90	0.47	0.03	5.70
Watermelon, 2004	9 (24)	0.91	-0.26	-0.46	1.38
Watermelon, 2005	11 (55)	0.81	-0.03	-0.26	2.34
Watermelon, 2007	6 (17)	0.94	0.28	0.02	1.63
Watermelon, 2008	10 (39)	0.72	0.38	-0.10	2.55
Watermelon, 2010	11 (45)	0.85	-0.02	-0.25	2.06

Supplementary references

1. Bommarco, R., Lundin, O., Smith, H.G. & Rundlof, M. Drastic historic shifts in bumble-bee community composition in Sweden. *Proc. R. Soc. B.* **279**, 309-315 (2012).
2. Bommarco, R., Marini, L. & Vaissière, B.E. 2012. Insect pollination enhances seed yield, quality and market value in oilseed rape. *Oecologia* **169**,1025-1032 (2012).
3. Holzschuh, A., Dormann, C.F., Tscharntke, T. & Steffan-Dewenter, I. Expansion of mass-flowering crops leads to transient pollinator dilution and reduced wild plant pollination. *Proc. R. Soc. B* **278**, 3444-3451 (2011).
4. Riedinger, V., Renner, M., Rundlöf, M., Steffan-Dewenter, I. & Holzschuh, A. Early mass-flowering crops mitigate pollinator dilution in late-flowering crops. *Landscape Ecology* **29**, 425-435 (2014).
5. Jauker, F., Diekötter, T., Schwarzbach, F. & Wolters, V. Pollinator dispersal in an agricultural matrix: opposing responses of wild bees and hoverflies to landscape structure and distance from main habitat. *Landscape Ecol.* **24**, 547–555 (2009).
6. Bartomeus, I. *et al*. Contribution of insect pollinators to crop yield and quality varies with agricultural intensification. *PeerJ PrePrints* 1:e184v1 http://dx.doi.org/10.7287/peerj.preprints.184v1 (2013)
7. Carré, G. et al. Landscape context and habitat type as drivers of bee diversity in European annual crops. *Agric. Ecosys. Environ.* **133**, 40–47 (2009).
8. Reemer, M. & Kleijn, D. Wild pollinators in apple and pear orchards in the Betuwe region, the Netherlands, in 2010 and 2011. Report number EIS2012-01 (2012). (In Dutch).
9. Móczár, L. The distribution of wild bees in the Lucerne fields of Hungary (Hymenoptera, Apoidea). *Annls. Hist.-Nat. Mus. Nat. Hung.* **53**, 451-461 (1961).
10. Tanács, L., Benedek, P. & Bodnár, K. Changes in the diversity and specific structure of lucerne pollinating wild bee assemblages (Hymenoptera: *Apoidea*) in Hungary in the past decades. *Növénytermelés* **57**, 341-357 (2008). (in Hungarian with English summary and table legends).
11. Rollin, O. *et al.* Differences of floral resource use between honey bees and wild bees in an intensive farming system. *Agric. Ecosys. Environ*. **179**, 78–86 (2013).
12. Le Féon, V. *Insectes pollinisateurs dans les paysages agricoles : approche pluri-échelle du rôle des habitats semi-naturels, des pratiques agricoles et des cultures entomophiles.* PhD thesis, University of Rennes 1 (2010). (In French).
13. Pisanty, G., Klein, A.M. & Mandelik, Y. Do wild bees complement honeybee pollination of confection sunflowers in Israel? *Apidologie* **45**, 235-247 (2014).
14. Pisanty, G. & Mandelik, Y. Profiling crop pollinators: life-history traits predict habitat use and crop visitation by Mediterranean wild bees. *Ecol. Appl.* **25**, 742-752 (2015).
15. Winfree, R., Williams, N.M., Gaines, H., Ascher, J. & Kremen, C. Wild bee pollinators provide majority of crop visitation across land use gradients in New Jersey and Pennsylvania, USA. *J. Appl. Ecol.* **45**, 793-802 (2008).
16. Cariveau, D.P., Williams, N.M., Benjamin, F.E. & Winfree, R. Response diversity to land use occurs but does not consistently stabilise ecosystem services provided by native pollinators. *Ecol. Lett*. **16**, 903-911 (2013).
17. Benjamin, F.E., Reilly, R.J. & Winfree, R. Pollinator body size mediates the scale at which land use drives crop pollination services. *J. Appl. Ecol.* **51**, 440–449 (2014)

18. Winfree, R., Williams, N.M., Dushoff, J. & Kremen, C. Native bees provide insurance against ongoing honey bee losses. *Ecol. Lett.* **10**, 1105-1113 (2007).
19. Mackenzie, K.A. & Averill, A.L. Bee (Hymenoptera: Apoidea) diversity and abundance on Cranberry in Southeastern Massachusetts. *Ann. Entomol. Soc. Am.* **88**, 334-341(1995).
20. Adamson, N.L., Roulston, T.H., Fell, R.D. & Mullins, D.E. From April to August—wild bees pollinating crops through the growing season in Virginia, USA. *Environ. Entomol*. **41**, 813-821 (2012).
21. Courcelles, D.M.M., Button, L. & Elle, E. Bee visit rates vary with floral morphology among highbush blueberry cultivars (*Vaccinium corymbosum* L.). *J. Appl. Ent.* **137**, 603-701 (2013).
22. Greenleaf, S.S. & Kremen, C.Wild bee species increase tomato production and respond differently to surrounding land use in Northern California. *Biol. Conserv*. **133**, 81-87 (2006).
23. Klein, A.M., Steffan-Dewenter, I. & Tscharntke, T. Fruit set of highland coffee increases with the diversity of pollinating bees. *Proc. R. Soc. B.* **270**, 955-961 (2003).
24. Klein, A.M., Steffan-Dewenter, I. & Tscharntke, T. Pollination of *Coffea canephora* in relation to local and regional agroforestry management. *J. Appl. Ecol*. **40**, 837–845 (2003).
25. Howlett, B.G., Walker, M.K., Newstrom-Lloyd, L.E., Donovan, B.J. & Teulon, D.A.J. Window traps and direct observations record similar arthropod flower visitor assemblages in two mass flowering crops. *J. Appl. Entom.* **133**, 553-564 (2009).
26. Carvalheiro, L.G. et al. Natural and within-farmland biodiversity enhances crop productivity. *Ecol. Let.* **14**, 251-259 (2011).
27. Ricketts, T.H., Daily, G.C., Ehrlich, P.R. & Michener, C.D. Economic value of tropical forest to coffee production. *Proc. Natl. Acad. Sci. USA* **101**, 12579–12582 (2004).
28. Jha, S. & Vandermeer, J.H. Contrasting bee foraging in response to resource scale and local habitat management. *Oikos* **118**, 1174-1180 (2009).
